# Stable and programmable formation of DNA nanostructures via inter-strand crosslinking by assembly-triggered oxanine and amine linkage

**DOI:** 10.1093/nar/gkag638

**Published:** 2026-06-22

**Authors:** Jae Eon Lee, Eui Kyoung Jang, Ryeo Gang Son, Jeehyun Park, Seung Pil Pack

**Affiliations:** Department of Biotechnology and Bioinformatics, Korea University, Sejong-Ro 2511, Sejong 30019, Republic of Korea; Department of Biotechnology and Bioinformatics, Korea University, Sejong-Ro 2511, Sejong 30019, Republic of Korea; Department of Biotechnology and Bioinformatics, Korea University, Sejong-Ro 2511, Sejong 30019, Republic of Korea; Department of Biotechnology and Bioinformatics, Korea University, Sejong-Ro 2511, Sejong 30019, Republic of Korea; Department of Biotechnology and Bioinformatics, Korea University, Sejong-Ro 2511, Sejong 30019, Republic of Korea

## Abstract

DNA’s programmable self-assembly makes it a valuable material for nanoscale applications, yet the reversible nature of nucleobase pairing limits the stability of assembled DNA structures, even under physiological conditions. Introducing covalent bonds between DNA strands after assembly offers a promising solution, but these bond-forming reactions must be controlled spatially and temporally. In this study, we explored oxanine (Oxa) as a novel, assembly-dependent crosslinker to address this need. The electrophilic C2 carbonyl of Oxa serves as the reactive site, undergoing a proximity-driven reaction with an amine group, enabling assembly-triggered covalent linkage between Oxa- and amine-bearing DNA strands. The exceptional stability of Oxa in aqueous solutions, combined with its proximity-specific reaction with amines, makes it an ideal approach for stable and programmable formation of strand-crosslinked DNA structures. We demonstrated the linkage of Oxa-modified and amine-modified strands across various DNA motifs, including duplexes, Y-shapes, and T-motif tiles. The assembly-triggered Oxa–amine (At-ON) linkage is compatible with diverse DNA structures, enhancing the stability of assembled DNA nanomaterials and devices.

## Graphical Abstract

**Graphical Abstract:**
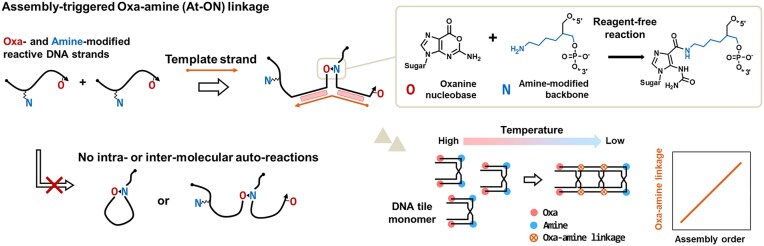

## Introduction

DNA, a biomaterial in nature which contains genetic information, has been re-evaluated as a useful building material [1]. It provides the ability for programmable self-assembly in nanoscale applications. These applications vary from logical interfaces that connect organic and inorganic materials to precise nanodevices based on blueprints [2–5]. The design freedom of DNA materials gives them tremendous potential. By following the rules of thermodynamics in base pairing, DNA can form functional composites that have both static and dynamic properties, allowing for numerous innovative nanodevices to be developed [6–9]. However, the reversible nature of base pairing interactions can also limit their applicability in dissipative systems under denaturing conditions. To address this issue, researchers have been actively studying ways to increase the stability of the self-assembled DNA platforms [9–13].

One promising approach is to introduce covalent bonds for inter-connections of participant strands within the assembled DNA structure. The first trial was the enzymatic method [14–18], where the ligase finds nicks in the DNA structure and seals them, allowing the fragments to join together in a chain in the structure. The issue with this method is that the ligase is sensitive to the local formulation of the DNA structure surrounding the target site [19, 20]. If the nick is concealed inside complex structures, the ligase would not be able to access it. Since most DNA structural designs rely on the multiple crossover motif that generates the vertex, a more universal approach is necessary in the field of structural DNA design.

Several enzyme-free inter-strand crosslinking strategies have been developed to enhance the structural stability of DNA assemblies through covalent bond formation [9, 10]. These strategies primarily rely on DNA-templated chemistry, where Watson–Crick hybridization brings reactive functional groups into close proximity. This proximity-acceleration principle facilitates bond formation by increasing the local concentration of reactants rather than relying on external chemical activation [21–25]. While this principle has been successfully applied to template-directed ligations and inter-strand crosslinking, established approaches such as copper-catalyzed azide–alkyne cycloaddition (CuAAC) and thymine–thymine (T–T) photochemical dimerization face significant compatibility issues. CuAAC requires an exogenous Cu(I) catalyst that is cytotoxic and difficult to integrate into a stepwise assembly, while T–T dimerization depends on UV irradiation, which risks off-target photodamage to the DNA nanostructures. Therefore, it is worth considering an approach that embeds reactivity directly into the assembly process itself, where it remains dormant until hybridization occurs and requires neither external catalysts nor exogenous energy inputs. Such a system would enable stable, sequential crosslinking across interconnected DNA structural motifs without the risk of collateral damage, and would represent a meaningful advance beyond the proximity-acceleration principle. A comparative overview of these and other major DNA inter-strand connection strategies is provided in Supplementary Table S1.

To ensure that anucleic acid is maximally effective in nanofabrication, it is crucial to identify the requirements of an ideal chemical crosslinker. These include: (i) no exogenous activating agent or co-catalyst to avoid unpredictable chemical damage to DNA strands or by-product formation; (ii) a slow and one-step reaction to mediate the crosslinks even in physiological conditions compatible with the DNA self-assembly process; and (iii) a DNA assembly-triggered reaction that can prevent unwanted (random) crosslinking within the participant DNA strands and allow the crosslink to follow the intended assembly order of interconnected DNA strands. We propose an ideal method for crosslinking assembled DNA structures using oxanine (Oxa) as a kinetically inert and proximity-dependent crosslinker, the reactivity of which is suppressed under non-assembly conditions but is activated exclusively upon proximity driven by DNA self-assembly.

**Scheme 1.**
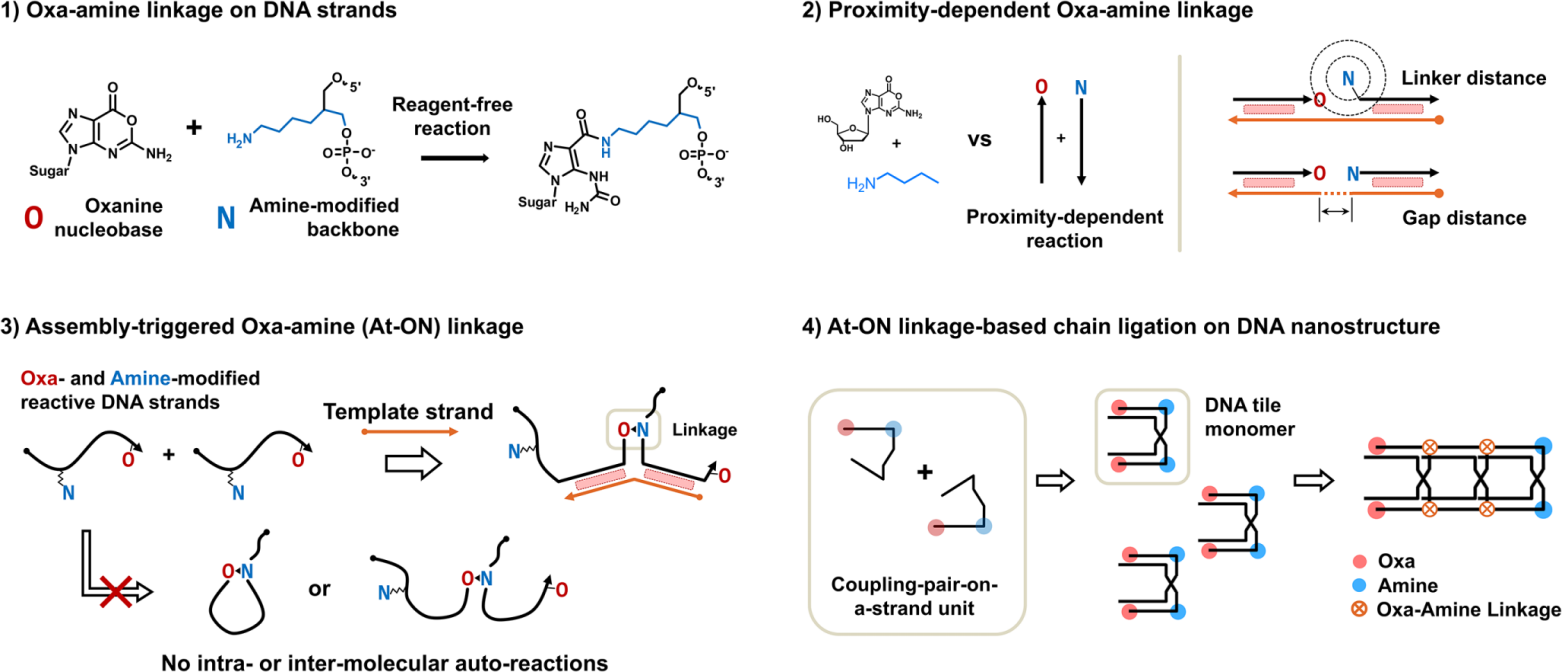
Assembly-triggered Oxa–amine (At-ON) linkage and its design for DNA nanostructure assembly. The Oxa–amine linkage forms between an Oxa nucleobase and an amine-modified DNA backbone via a reagent-free reaction. The reaction is proximity dependent, proceeding selectively under templated conditions; crosslinking efficiency is further governed by linker and gap distances between the reactive groups. Upon template strand-directed assembly, Oxa- and amine-modified reactive DNA strands undergo selective At-ON linkage formation with no intra- or inter-molecular auto-reactions. A coupling-pair-on-a-strand unit enables chain ligation across DNA tile monomers, generating extended DNA nanostructures stabilized by Oxa–amine linkages at the junctions.

Previously, we reported Oxa, a modified nucleobase with an electrophilic reactive site, as a novel crosslinker for covalently linking oligodeoxynucleotides with amine-functionalized molecules, including chemicals and peptides [26, 27]. Oxa is a lesion formed by the nitrosative oxidation of guanine (Gua) [28, 29], which bears an *O*-acylisourea (a carbodiimide-attached carboxylate-like group) within the nucleobase ring (Scheme 1; Supplementary Fig. S1A). The investigation of Oxa’s reaction characteristics was initiated based on its unique biological roles—a series of *in vitro* experiments revealed that Oxa can form DNA adducts with DNA-binding small molecules and proteins, via covalent-bond formation with amine and thiol groups. The formation of these adducts was found to impede the processability of helicase and polymerase, with the extent of inhibition being size dependent, thereby demonstrating potent biological genotoxicity [30, 31]. In the development of conjugation systems, Oxa offers an opportunity for an exogenous-reagent-free mode in DNA-based applications [26, 27]. Oxa is exceptionally stable in aqueous solutions, even under high pH conditions [32], making it an ideal moiety for maintaining a kinetically inert reactivity before the crosslink reactions (Supplementary Fig. S1).

In this study, we characterized Oxa as an assembly-dependent crosslinker in terms of proximity dependency and established the assembly-triggered Oxa–amine (At-ON) linkage system, including the design of coupling-pair-on-a-strand (Oxa and amine on the same strand) enabling sequential chain ligation, across a range of DNA structural motifs (Scheme 1). The advantages of the kinetically inert state of Oxa reactivity and the spatial proximity-driven reaction mediated by the DNA self-assembly were also characterized in addition to the concentration-dependent reaction. This assembly-mediated DNA hybridization forms an amide bond between Oxa and the amine group on proximity-localized DNA strands. Oxa–amine linkages were analyzed for the crosslinks within various types of DNA structural motifs and finally employed for the assembly-triggered crosslinking of DNA nanostructures. As expected, Oxa–amine linkage showed a negligible level of auto-self- or random crosslink reactions, which are unwanted intra- or inter-strand covalent formations prior to the DNA self-assembly event. As a result, the At-ON linkage method has been compatibly employed for programmable and stable formation of DNA nanostructures (e.g. T-motif tiles).

## Materials and methods

### Materials

The 2′-deoxynucleoside form of Oxa (dOxo) for stability analysis and OTP (oxanosine triphosphate) for preparation of Oxa-modified DNA strands were converted from deoxyguanosine (cat. # D7145, Sigma-Aldrich, St. Louis, MO, USA) and GTP (G8877, Sigma-Aldrich), respectively, using the nitrosative oxidation method [33, 34]. Terminal deoxynucleotidyl transferase (TdT) was purchased from New England Biolabs (M0315, NEB, Ipswich, MA, USA). Oxa–amine reaction buffers were prepared using MES (M3671, Sigma-Aldrich) and HEPES (H3375, Sigma-Aldrich). DNA strands (Supplementary Table S2) were prepared using a commercial service as follows. For the amino modification at the 5′ end of DNA strands, a six-carbon (C6) and 12-carbon (C12) aliphatic spacer with a primary amine were used (IDT Co., Coralville, IA, USA). For the amino modification in the middle of the phosphate backbone, int Uni-link™ was applied (IDT Co.). Non-modified oligonucleotides were provided by Cosmogenetech Co.(Seoul, Republic of Korea). Options for aliphatic spacers are limited: C6 is available for amine modifications at both internal backbone and terminal positions (5′ or 3′ end), whereas C12 is only available for terminal amine modifications (Supplementary Fig. S2; Supplementary Table S2). Consequently, end-to-end DNA linkage reactions utilized amines with C12 and C6 linkers (Supplementary Fig. S2C). In contrast, Oxa–amine linkages in overhang motifs, such as #3 and T-motif tiles, which require internal backbone amine modifications, used a C6 linker.

### Preparation of oxanine-modified DNA strands

To obtain the Oxa-modified DNA strands, we applied the TdT-based single-stranded DNA (ssDNA) end-labeling method as described previously [35, 36]. Briefly, the target DNA strands (3 μM; final concentration, same as below), OTP (150 μM), MgCl_2_ (10 mM), CoCl_2_ (0.25 mM), TdT (0.8 U μl^−1^), and TB buffer (100 mM Tris with 150 mM borate, pH 8.0) were mixed and incubated at 37°C for 60 min. To stop the reaction, 4 μl of 0.3 M EDTA was added per 25 μl of the reaction mixture, then heated at 90°C for 10 min. The reaction product was concentrated and residual substrates were eliminated by a 3 kDa MWCO (molecular weight cut-off) centrifugal filter after 30 kDa filter treatment (Amicon™, Merck KGaA, Darmstadt, Germany). Single incorporation of OTP into the substrate strand by TdT was verified by MALDI-TOF MS (matrix-assisted laser desorption ionization time-of-flight mass spectrometry) analysis: the measured mass increment of the extended product (F20dN.rO1, Δm = +340.6 Da relative to F20dN) is consistent with addition of one Oxa nucleoside monophosphate residue with concomitant release of pyrophosphate, confirming stoichiometric single-nucleotide extension (Supplementary Fig. S3).

### Reactivity and stability analysis of oxanine

For the reactivity test, dOxo (1 mM) was incubated with 20 mM butylamine (BA) (90 893, Sigma-Aldrich) in HEPES (300 mM, pH 8.4) buffer at 24°C. For the stability analysis, dOxo (3 mM) was incubated in distilled water (DW), MES (300 mM, pH 7.4), and HEPES (300 mM, pH 8.2) at 24°C. The reaction mixture was sampled over time and measured using high-performance liquid chromatography (HPLC; Young Lin Co., Korea, YL9100 system); column = ULTRON VX-ODS 150 × 4.5 mm, 5 μm; gradient buffer system = 0% ACN (acetonitrile) in 0.1% TFA (trifluoroacetic acid) at 0 min to 20% ACN in 0.1% TFA at 20 min, 1 ml min^−1^ flow rate; detection wavelength = 260 nm.

### Reaction sustainability of oxanine-labeled DNA strand

To determine the loss of reactivity by hydrolysis, the Oxa-labeled DNA strand (*a1{O}*) was incubated under varying pH conditions, then the residual reactivity of Oxa was assessed via its reaction with excess amine molecules (spermine). Specifically, 0.5 μM Oxa-labeled DNA strand was incubated in 5 mM buffers (MES pH 6.4, MES pH 7.4, and HEPES pH 8.4), followed by treatment with 33 000 times excess spermine. The residual reactivity was calculated by measuring the ratio FI_products/(FI_products + FI_remains) from the PAGE (polyacrylamide gel electrophoresis) separation result (Supplementary Fig. S4).

### Kinetic analysis of oxanine–amine reactions

**Figure 1.**
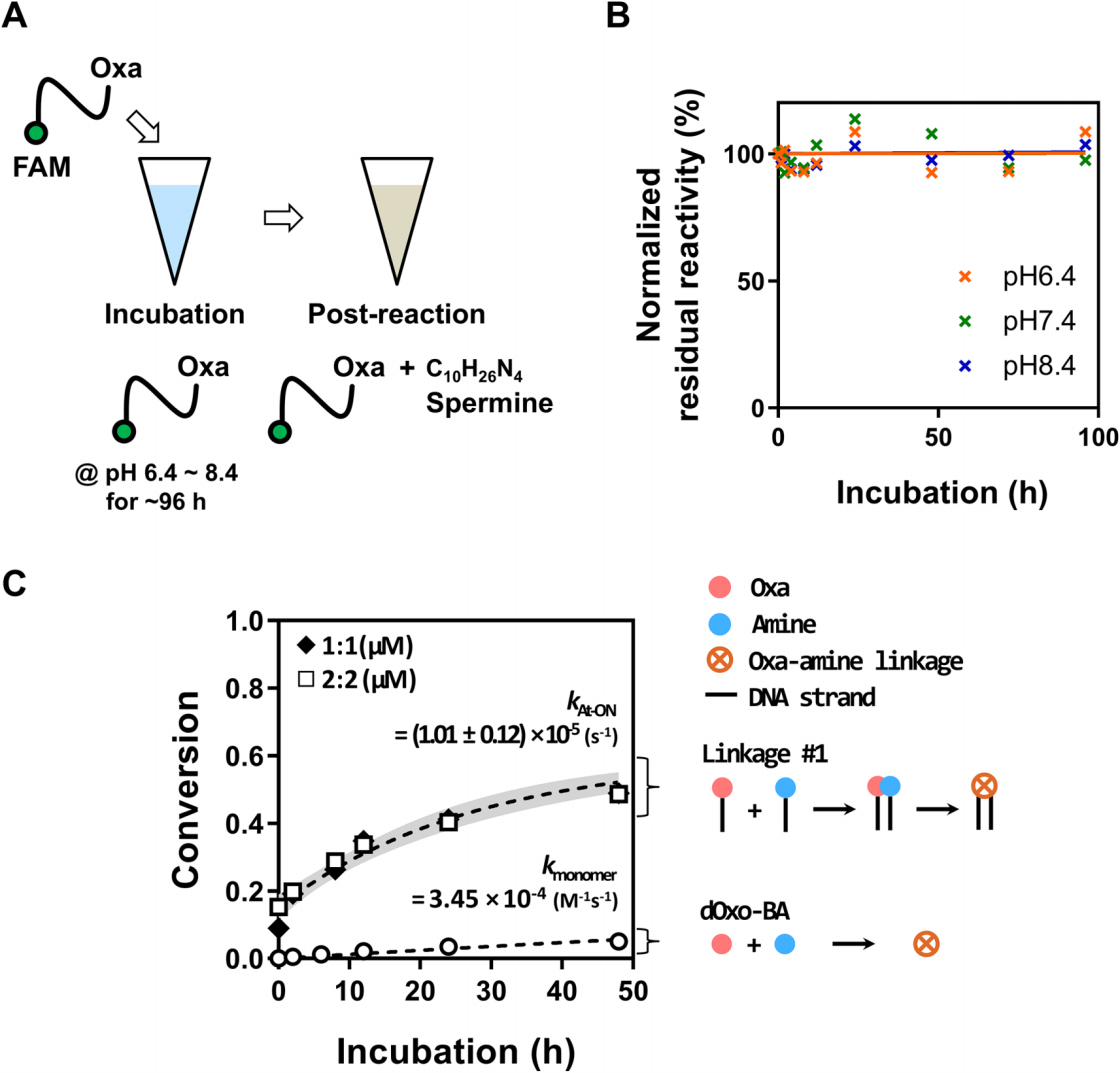
Reaction sustainability of Oxa-labeled DNA strands under varying pH conditions and kinetics of DNA hybridization-directed Oxa–amine reactions. (**A** and** B**) The DNA strand *a1{O}* (Oxa at the 3′ end) was incubated at pH 6.4–8.4; residual reactivity was assessed by reaction with excess spermine. (**C**) The observed rate constant was determined for a monomer-scale reaction (dOxo and BA, 1 mM each) and a DNA hybridization-directed At-ON linkage [linkage #1, 2 μM (white square) and 1 μM (black diamond) per strand]. The two reactions at different concentrations yielded statistically indistinguishable conversion profiles (*F*-test, *P* = 0.457), with the common fitted curve shown with the 95% confidence interval (gray). The value of *k*_At-ON_ is reported as the best-fit value ± standard error. Supplementary Table S2 provides the sequences and abbreviations of all DNA strands used in this study.

For the monomer-scale reaction, 1 mM dOxo with 1 mM BA was incubated in 300 mM HEPES (pH 8.4) at 24°C. The product was measured using HPLC. The monomer-scale reaction (dOxo and BA) follows second-order kinetics [27], yielding *k*_monomer_ (M^−1^ s^−1^). The effective molarity of the DNA-templated reaction was subsequently calculated as the ratio of *k*_At-ON_ to *k*_monomer_ (see below). For the DNA-templated reaction, 1 μM and 2 μM of each DNA strand for Oxa–amine linkage #1 were incubated in 50 mM HEPES (pH 8.4) containing Mg(OAc)_2_ (20 mM) at 24°C. Product conversion was quantified by fluorescence imaging of PAGE-separated samples. Since the initial strand concentration (1–2 μM) greatly exceeds the duplex *K*_d_ (low nM range), the DNA-templated reaction was treated as first-order. The conversion curves at both concentrations were fitted with a first-order exponential decay to obtain *k*_At-ON_ (s^−1^) (Fig. 1) Effective molarity in At-ON was calculated by dividing *k*_At-ON_ (s^−1^), intra-reaction, by *k*_monomer_ (M^−1^ s^−1^), inter-reaction [37, 38].

### Assembly-triggered oxanine–amine linkages

For non-T-motif-based assembly-triggered Oxa–amine linkages, we made a mixture that contains an Oxa-modified strand (2 μM), an amine-modified strand (2 μM), and a template strand (if needed) (2 μM) in HEPES (50 mM, pH 8.4) buffer containing Mg(OAc)_2_ (20 mM). The mixture was incubated at 37°C over time. For T-motif assembly-triggered Oxa–amine linkages, a final 0.6 μM of four strands which consist of the T-motif junction were added in HEPES (50 mM, pH 8.4) buffer containing Mg(OAc)_2_ (20 mM). Then, the mixture was heated to 90°C for 5 min and cooled to a specific reaction temperature for further examination.

### Oxanine–amine linkage for multimerization of T-motif tiles

To perform the crosslinking of T-motif tiles driven by multimerization, we mixed Oxa-modified T-motif tile strands (L+*{N,O}* and L−*{N,O}*, Supplementary Table S2) (1 μM) in HEPES (50 mM, pH 8.4) buffer containing Mg(OAc)_2_ (20 mM). The mixture was heated at 90°C for 5 min, and cooled down to 20°C, after which it was incubated further at 20°C for 72 h. For the stability test, inhibition tiles (12.5 nM to 0.8 μM) and the products (0.2 μM) were mixed in HEPES (50 mM, pH 8.4) buffer containing Mg(OAc)_2_ (20 mM) and incubated further at 30°C for a maximum of 3 h.

The integrity of the multimer was determined by quantifying the signal up to a length of 300 bp using a size marker. Subsequently, a dose–response curve was adjusted using the following equation [39]:


(1) \begin{eqnarray*}
\textit{Integrity}\left( \% \right) = \frac{{100}}{{1 + {{{\left( {\frac{{IC50}}{R}} \right)}}^n}}}
\end{eqnarray*}

Where, IC50 is the concentration of R at which the integrity is reduced by half; R represents the ratio of inhibitors used per tile; *n* is the Hill slope.

### Polyacrylamide gel electrophoresis separation and quantification

The reaction samples were separated by denatured PAGE in a 7 M urea solution (10% gel) as the default method. If native PAGE was used as an alternative, it is specified in the figure legend. The separated samples were quantified using a fluorescence image analyzer (LAS 4000, General Electric Co.) in the detection mode for the FAM dye (495 nm/520 nm). Product (%) was defined as FI_product/(FI_product + FI_remaining) × 100, where FI denotes the fluorescence intensity of each band from denaturing PAGE. Linkage (%) in Figs 3 and 5 is defined analogously, with linkage referring specifically to the product of the Oxa–amine reaction.

## Results and discussion

### Reaction properties of oxanine and oxanine-modified DNA strands

We investigated the stability of dOxo, the 2′-deoxynucleoside form of Oxa, in various solutions including DW, HEPES buffer at pH 7.4, and HEPES buffer at pH 8.2, where some Oxa undergoes hydrolysis and is converted to xanthine (Xan), a non-reactive compound [40]. Using HPLC, we quantified the remaining amount of dOxo after 100 h of incubation. The results showed that 90, 71, and 28% of dOxo remained in the solutions of DW, neutral pH, and basic pH, respectively (Supplementary Fig. S1B). From the exponential decay curve, the half-life of dOxo was determined in DW, HEPES pH 7.4, and HEPES pH 8.2. These results suggest that Oxa with *O*-acylisourea in the nucleobase ring exhibits exceptional stability compared with other catalyst-activated carboxylate forms. In particular, the activation span of the EDC–NHS [1-ethyl-3-(3-dimethylaminopropyl)carbodiimide–*N*-hydroxysuccinimide) compound in neutral pH solution is <4 h [41], whereas Oxa has been shown to maintain stability (almost 200 h at neutral pH) beyond this time frame.

Moreover, the long-term sustainability of Oxa reactivity for high pH was shown to dramatically increase when Oxa was loaded at the end of DNA strands (Fig. 1A, B; Supplementary Fig. S4). Enhanced sustainability is likely to be due to steric and/or electrostatic shielding of the reactive carbonyl by the adjacent DNA backbone and base-stacking interactions. The superior stability of Oxa reactivity under relatively basic pH conditions provides a distinct advantage in promoting covalent bond formation between Oxa and amine groups, as the nucleophilicity of amines is enhanced at higher pH levels.

To investigate the efficiency of the reaction between Oxa and amine under varying pH solutions, we incubated dOxo with BA at 24°C over time in buffers of pH 6.4, 7.4, and 8.4 (Supplementary Fig. S1C). The reaction product (Oxa–BA) was analyzed using HPLC (Supplementary Fig. S1D). The Oxa–amine reaction relies on the pH range. In the buffer solution with pH 8.4, the initial production rate was 8.8 times higher than at pH 7.4. Conversely, the reaction was hindered at pH 6.4. This can be attributed to the increase in nucleophilicity of the primary amine as the reaction pH increases [42]. Additionally, the monomer-scale Oxa–amine reaction rate was influenced by the concentration of reaction groups, as it follows second-order kinetics [27], yielding an intermolecular rate constant *k*_monomer_ (M^−1^ s^−1^). In contrast, when Oxa and amine are brought into close proximity by DNA hybridization, the reaction becomes effectively first-order (see the kinetic analysis below). When the reaction was conducted with a 1:1 molar ratio of Oxa to BA, the reaction rate decreased by 20 times compared with conditions where 20 mM BA was used, showing only 5% of conversion after 48 h in the reaction condition.

When Oxa reacts with an amine molecule (BA) at the monomer scale, the reaction proceeds under catalyst-free conditions in the crosslinking process. Notably, it shows exceptional long-term stability even against the base-promoted hydrolysis at high pH. The reaction rate between Oxa and amine is relatively slow, but can be controlled by adjusting the molar ratio of the amine. Unless Oxa and amine groups are brought into close proximity, Oxa maintains the kinetically inert reactivity in the mild conditions. The result of employing charge-to-charge interactions between an Oxa-modified strand and a cationic peptide (10 mer lysine) provides preliminary evidence that the crosslinking in which Oxa is involved could be controlled by localizing the reactive groups through macromolecular interactions (Supplementary Fig. S5). Oxa could be an ideal reactive moiety for crosslinking reactions that occur over a long period with minimal side reactions under physiological conditions, when triggered by controlled macromolecular interactions, such as DNA complementation or hybridizations.

### Assembly-triggered oxanine–amine linkage in duplex and Y-shaped strands

We investigated the reaction between Oxa and amine for the case of a DNA duplex. The crosslinking reaction can be accelerated by increasing the proximity of the amine group to Oxa via the sequence-dependent affinity between two complementary DNA strands (Fig. 1C). Conversely, if sequence complementarity is excluded, the reaction rate between Oxa and amine molecules would be substantially low, as observed in the reactions between the single molecules (Fig. 1C; Supplementary Fig. S1E). In fact, the introduction of Oxa and amine at a specific position within each participating DNA strand enables the construction of inter-connected DNA structures with high specificity through the assembly process. When two complementary strands were prepared by incorporating Oxa on the 3′ end of one DNA strand and an amine group on the 5′ end of the other DNA strand, the reaction occurs readily.

To quantify the kinetic advantage conferred by DNA hybridization, we measured the observed rate constants for both the monomer-scale reaction (dOxo and BA, 1 mM each) and the DNA-templated At-ON linkage (#1) at two strand concentrations (1 μM and 2 μM per strand). Since the initial strand concentration greatly exceeds the duplex *K*_d_ (low nM range), the DNA-templated reaction was treated as first-order. The conversion curves at the two concentrations were statistically indistinguishable (*F*-test, *P* = 0.457 > 0.05), confirming that the fractional conversion is independent of the initial strand concentration, consistent with first-order kinetics (Fig. 1C). The effective molarity, calculated as the ratio of *k*_At-ON_ (s^−1^) to *k*_monomer_ (M^−1^ s^−1^), was ~14 000-fold higher for the DNA-templated reaction relative to the monomer-scale reaction, demonstrating the pronounced proximity effect of duplex formation on the Oxa–amine reaction.

**Figure 2.**
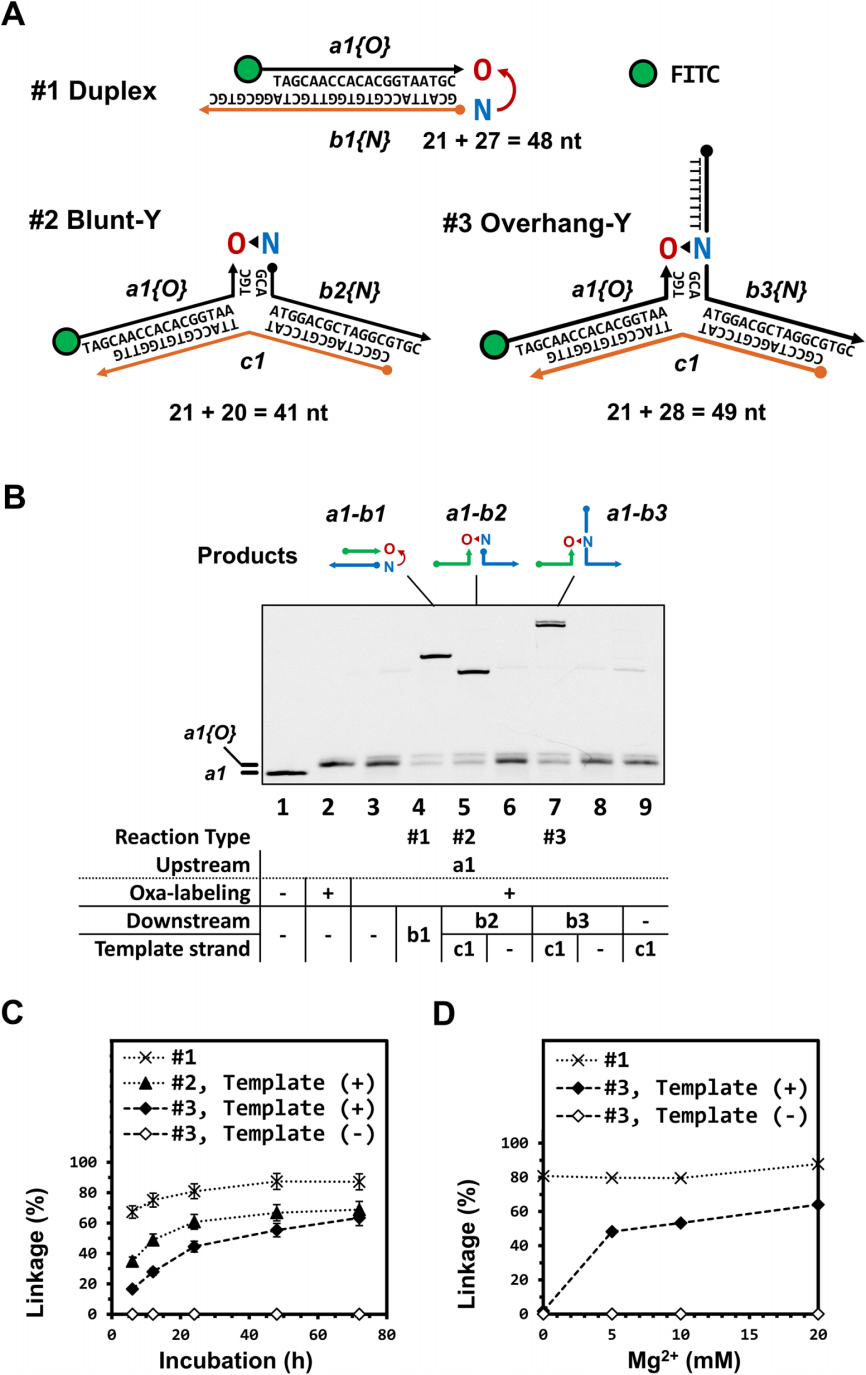
DNA motif models for At-ON linkages. (**A**) #1 is a reaction on a complementary duplex, #2 is a templated reaction on blunt-Y, and #3 is a templated reaction on an overhang-Y. (**B** and **C**) Three types of Oxa–amine reactions were conducted with or without a template strand. (**D**) The magnesium dependency of #3. (B–D) After visualizing the results using denaturing PAGE, the intensity of FITC (fluorescein isothiocyanate) on the strand (*a1*) was quantified. The error bars indicate the standard deviation range (*n* = 3).

To determine the specificity of Oxa–amine linkage triggered by the assembly, the intended interaction process among the DNA strands, we prepared Y-shaped forms, in which the linkages are initiated by adding a guide DNA strand (Fig. 2A). The template strand facilitates the assembly of two reaction strands; the DNA strand modified with Oxa at its 3′ end and the DNA strand modified with amine at its 5′ end (for #2) or the internal backbone (for #3) for the purpose of proximity-induced crosslinking, resulting in formation of an amide bond between Oxa and the amine group. The Y-shaped blunt (#2) and overhang (#3) forms underwent the linkage reaction in HEPES buffer (pH 8.4) for 48 h at 37°C with or without the template *c1*. Upon PAGE separation (Fig. 2B), each crosslinked product with a size of 41 nt (for #2) and 49 nt (for #3) was observed when the template *c1* was included in the reaction (lanes 5 and 7).

The product size of #3 (49 nt) is similar to that of #1 (48 nt); however, the mobility of #3 on the PAGE gel was slower than expected based on its actual size because of its steric complexity. The At-ON linkages were found to be highly specific in the presence of the template strand, since no observable crosslinked products were detected when the template strand was absent (lanes 6 and 8). The efficiency of the At-ON linkages was ranked from high to low as #1, #2, and #3, which were 87, 67, and 55%, respectively, after incubating for 48 h (Fig. 2C; Supplementary Table S3, which lists all the conformations of Oxa–amine linkages, their IDs, and the reaction conditions). Under nucleophile-rich conditions, the reactivity of the incorporated Oxa was confirmed to be nearly 100% (Supplementary Fig. S5D). These results demonstrate that the duplex form (#1), which employs a flexible C12 aliphatic amine spacer, achieves > 80% yield, whereas overhang and T-motif configurations (#2 and #3), relying on internal backbone amine modifications with a shorter C6 linker, show comparatively lower yields, as also demonstrated by the gap-dependent reactivity in Supplementary Fig. S6. The primary constraints are the restricted conformational freedom of the C6 amine linker and the steric and conformational demands imposed by architectures beyond a simple duplex, which reduce the probability of a productive Oxa–amine encounter. Optimization of the amine linker length and flexibility for non-duplex motifs is therefore identified as a key direction for improving crosslinking yields in future work.

The efficiency of At-ON linkages is also influenced by the presence of magnesium ions (Fig. 2D). In the absence of magnesium ions, the At-ON linkage results were not observed for #3, despite the cooperative behavior of the template strand. However, there is no significant difference in the reaction of the fully matched duplex (#1) without magnesium ions compared with the reaction in the presence of magnesium ions. Since the magnesium ion increases the stability of base pairing [43], the reaction failure of #3 can be attributed to the less stable stem region [44], which consists of only 3 bp, at the reaction temperature. These results underline the importance of ensuring the stability of local base pairing at the reaction site as well as providing the guide DNA.

**Figure 3.**
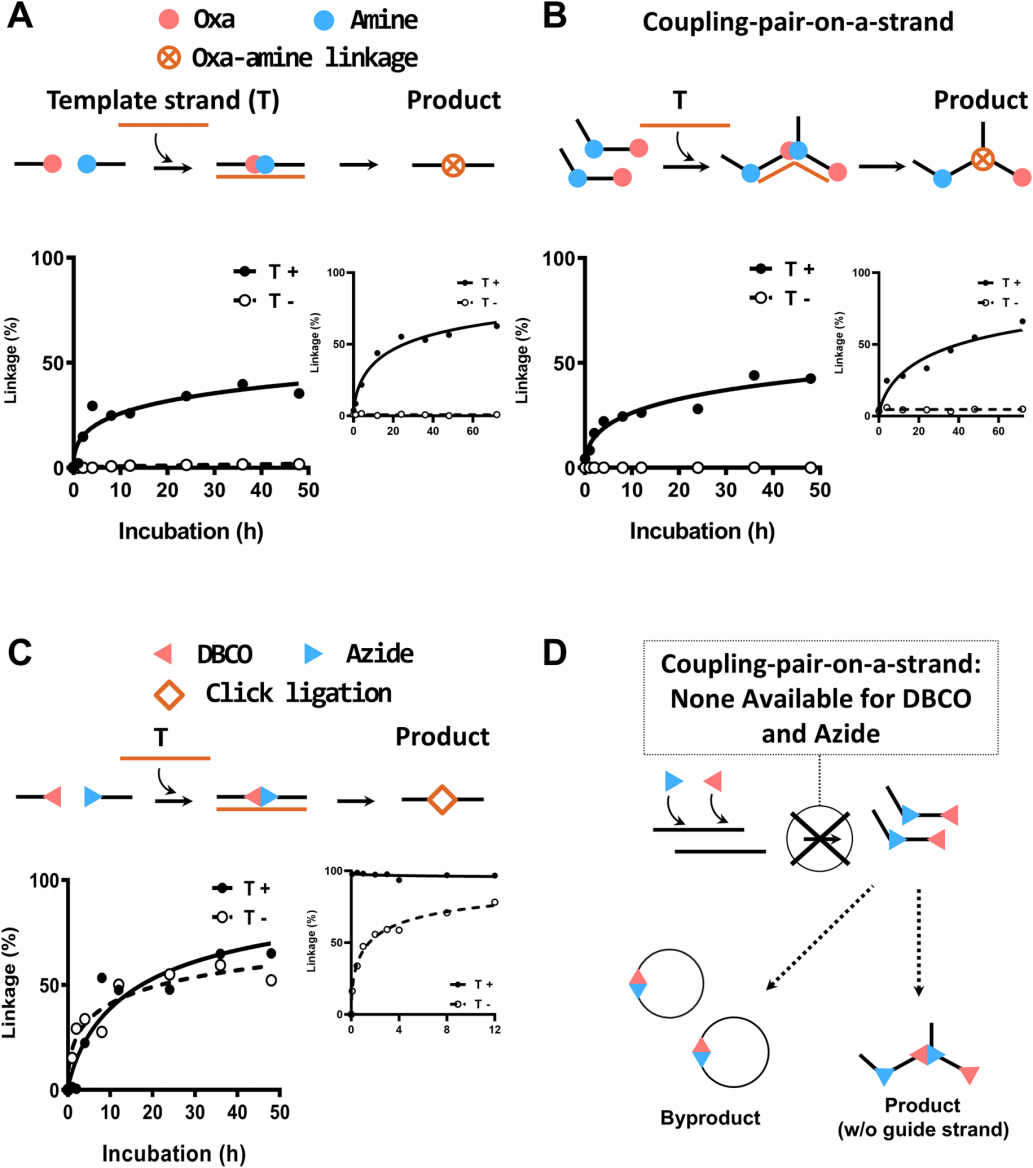
Controllability of the linkage reaction via the presence and absence of a template, demonstrating the coupling-pair-on-a-strand motif enabled by Oxa–amine chemistry. (**A**) The oxa-modified strand (*a2′{O}*) and amine-modified strand (*b1{N}*) were incubated with and without the template strand (*c6*). (**B**) The templated reaction was compared with the model system for the copper-free click reaction, using DBCO- and azide-modified strands (*a2′{D}* and *b1′{N3}*) with the template (*c6*). (**C**) At-ON linkages formed selectively as the coupling-pair-on-a-strand, where dual modifications (i.e. coupling pair) were present on the strand (*b1′{N,O}*) with the template (*c7*). (**D**) The equivalent coupling-pair-on-a-strand design is not achievable with DBCO–azide chemistry, as maintaining a non-reactive DBCO–azide configuration on the strand prior to template strand hybridization is chemically unfeasible; the two groups react without template strand control, precluding programmable on-strand crosslinking. Reactions were performed at pH 8.4 (standard conditions), and corresponding reaction yield plots under the optimized conditions for each system are shown in the upper-right corner. Quantification of the data was obtained from results of denaturing PAGE separation. Product (%) = FI_product/(FI_product + FI_remaining) × 100, where FI denotes fluorescence intensity of each band; this metric is equivalent to the Linkage (%) used in subsequent figures (see the Materials and methods for a full definition).

The kinetically inert electrophilicity of Oxa is essential for enabling proximity-driven reactions with amine groups during the self-assembly process, allowing an on–off toggle-like reaction mode based on the presence of a template strand. The assembly dependency of Oxa–amine linkages was compared with the commonly used exogenous-reagent-free conjugation method, the copper-free click reaction (Fig. 3). In time-course analysis, Oxa–amine linkages primarily occurred in the presence of the template strand (Fig. 3A). The longer strand formed by the templated Oxa–amine crosslink showed an increase in melting temperature by 6°C. This templated process is consistent in dual modifications, involving Oxa–amine coupling within a single strand (Fig. 3C). In contrast, the dibenzocyclooctyne (DBCO)–azide-mediated reaction produced similar levels of conjugation products regardless of whether the template strand was used or not (Fig. 3B). These results support that the Oxa–amine reaction, with its proximity-dependent reactivity, is more compatible with the stepwise process of DNA assembly formation. We additionally evaluated both reactions under optimized conditions. In the case of the DBCO–azide reaction, near-perfect conversion (∼100%) was achieved in the presence of the template-strand, but a substantial level of crosslink reaction (∼78%) was also observed even in the absence of the template-strand (Fig. 3C, right panel). This result highlights a fundamental limitation of DBCO –azide chemistry, in that the reaction proceeds constitutively, but cannot be effectively regulated by using the template strand. In contrast, our system, i.e. At-ON linkage, exhibits strict dependence on the presence of the template strand (Fig. 3A, B), thereby enabling precise control over the crosslinking events between strands while minimizing uncontrollable inter-molecular auto-reaction. Critically, the template strand dependence demonstrated above directly enables the design of a coupling-pair-on-a-strand unit, in which both Oxa and amine modifications reside on the same DNA strand and undergo selective crosslinking exclusively upon template strand hybridization (Fig. 3C).

**Figure 4.**
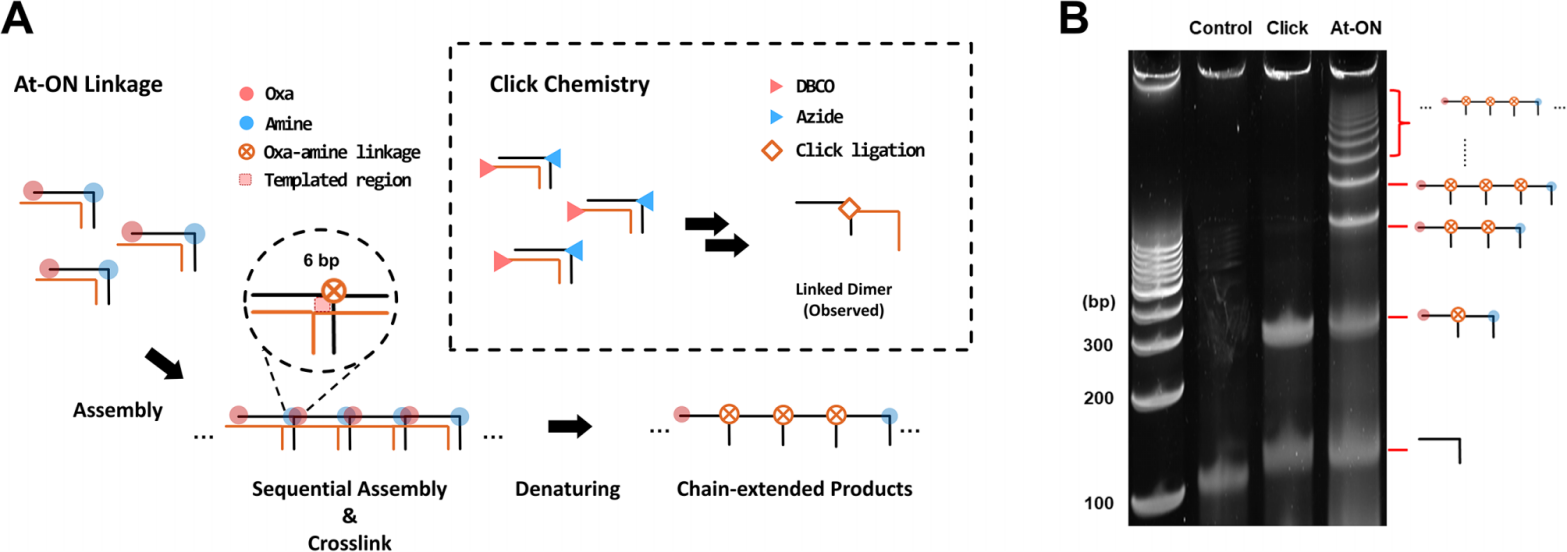
Sequential chain ligation via the coupling-pair-on-a-strand unit. (**A**) In At-ON (top), both Oxa and amine on a single strand enable self-propagating chain ligation upon template strand assembly. The DBCO–azide click reaction (dot box) requires separate strands for each reactive group. Both reactions were carried out under identical conditions, including the same sequences and reaction parameters, except for the reactive functional groups. (**B**) Denaturing PAGE confirms that At-ON produces higher-order chain-extended products, whereas click reaction yields only lower-order species (linked dimer).

This coupling-pair-on-a-strand design further enables sequential chain ligation, wherein each recruited strand introduces a fresh pair of reactive groups to propagate covalent linkages in a chain-like fashion, a capability absent in DBCO–azide click chemistry, which requires reactive partners on separate strands (Fig. 4). The structural consequence of this chain ligation capacity is further demonstrated in the context of T-motif tile multimerization (see Fig. 6)

The reaction characteristics of #1, #2, and #3 in template strand- and magnesium ion-dependent results indicate that Oxa–amine linkages can be triggered by both macromolecular interaction of DNA strands and optimal base pairing at the reaction site. Specifically, the Oxa–amine linkage on overhang structures such as #3 has great potential since the single strand domain allows additional sequence-based macromolecular interaction. We reorganized the interactive domain into the self-assembling unit for T-motif-based tile structure and evaluated At-ON linkages via an enhanced proximity-induced reaction that can be driven by base pairing of the junctional motif.

### Assembly-triggered oxanine–amine linkage in T-motif tiles: junction design

We investigated whether the At-ON linkage is effective for designing assembled DNA nanostructures and nanodevices, using T-motif tiles as a model system. The T-motif tile is a DNA architecture system to construct large size structures of DNA through assembly of various multimers using just two DNA strands [45]. However, the junction site of the T-motif is expected to be vulnerable in a denaturing environment because it relies on weak binding of 5–6 base pairs. First, we examined the results of Oxa–amine linkages at the junction of the T-motif, which was assembled in two parts. The assembly involves the hybridizations of the 3′ overhang template or 5′ overhang template with each target loop (Supplementary Figs S7A and S8). When using the 3′ overhang template (#4), Oxa-modified strand *a2* can directly attach to the loop domain of *c4* and react with an amine group in the middle of *b4*, resulting in *a2–b4* linkage product. In contrast, when using the 5′ overhang template, the *a2–b4* reaction was mediated via interaction of *d2* with the loop in *b4*.

It can be seen in Supplementary Fig. S7B that the linkages for both T-motif configurations were only found under the matched template strand involved in the reaction. However, there was a difference in the reactivity; the model with a 5′ overhang template (#5) was ~1.3 times more effective in the reaction compared with the model with a 3′ overhang template (#4) (Supplementary Fig. S7C). It is thought that the difference comes from the molecular orientation surrounding the reaction points. In the case of #4 (Supplementary Fig. S8A, B), Oxa and the amine group would meet at a cavity of a single DNA groove. However, the phosphate backbone crossing the cavity would restrict the degree of freedom of the amine group, because the amine group is immobilized on the inner strand of the loop structure. On the other hand, #5 (Supplementary Fig. S8C, D) permits a relatively wide space made up of two facing grooves, which may allow amine to encounter Oxa with flexibility.

**Figure 5.**
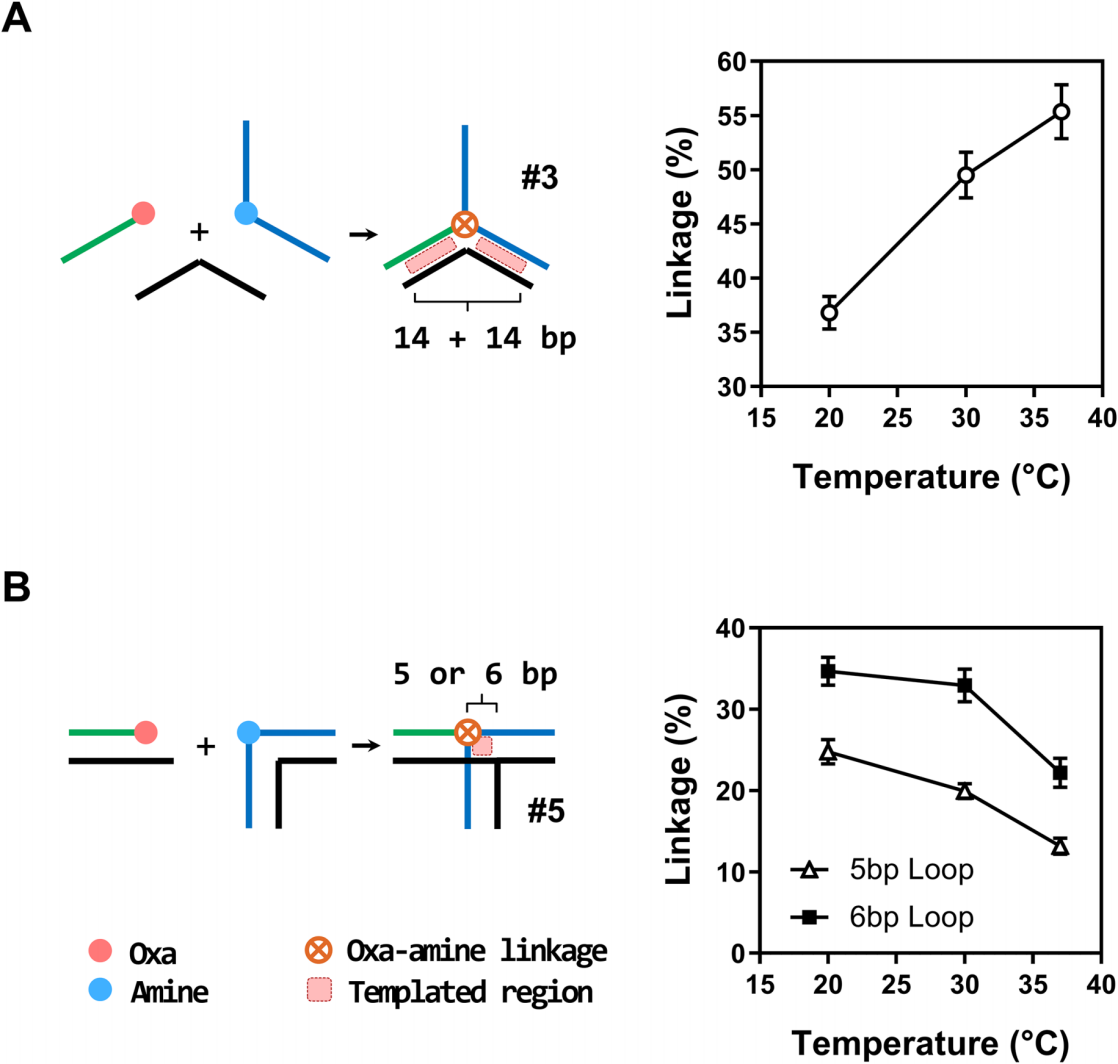
The trends in assembly-specific linkage production depend on the reaction temperature. (**A**) The efficiency of Oxa–amine linkage was evaluated in a 14 + 14 bp templated reaction and a T-motif that offers only 5–6 bp in a loop for assembly (**B**).

Next, we explored the dependency on temperature of the Oxa–amine linkages of T-motif tile assembly.Increasing temperature can speed up most chemical reactions as is established in collision theory [46]. When variable temperatures were applied to the Oxa–amine linkage supported by a long template strand (14 bp for each reactive DNA strand), crosslinking efficiency increased proportionally with reaction temperature (Supplementary Fig. S9C). To rationalize this observation, we performed a numerical simulation modeling the assembly-triggered reaction as A + B ⇌ AB → P, where the equilibrium population of the duplex intermediate is governed by the *T*_m_ of the binding domain. For the 14 bp template strand (*T*_m_ ~74°C), the simulation predicts increasing product formation with temperature in the experimental range, consistent with the experimental result (Fig. 5A; Supplementary Fig. S10A, B).

However, the results of the Oxa–amine linkages in T-motif tiles demonstrate that their crosslinking efficiency is inversely proportional to the reaction temperature (Fig. 5B; Supplementary Fig. S7B). Additionally, the long template–loop interaction with 6 bp shows ~1.4 times more effectiveness for the linkage than the short template–loop interaction with 5 bp. These results indicate that the assembly of T-motif tiles is based on a weak interaction with low thermal stability and, during the subsequent process of self-assembly of DNA strands, Oxa–amine linkages are initiated during the self-assembly process. Consistently, the same simulation predicts an inverse temperature dependence for a weak binding domain (*T*_m_ ~20°C, representative of the 5–6 bp T-motif junction), as the fraction of assembled duplex declines sharply above the*T*_m_ (Supplementary Fig. S10A, B). This agreement supports the interpretation that the observed inverse temperature dependence reflects the thermal instability of the junction rather than the intrinsic reactivity of Oxa, providing indirect evidence that At-ON crosslinking is assembly dependent.

### Oxanine and an amine group within one DNA strand for DNA architecture design

One of the promising strategies that increase the stability of DNA architecture dramatically comes from a topological approach such as forming interlocked strands [47], which include catenanes [48], rotaxanes [49], and knots [50]. In these structures, enzymatic ligation is commonly applied to seal the nick site for the template-directed intertwining structure. However, to achieve such a stabilized complex using non-enzymatic methods, both reaction groups, including at least one assembly-dependent crosslinker, should be incorporated within the same DNA strand. Moreover, these dual modifications do not affect the assembly process of all the participing DNA strands, i.e. the self-assembling units are allowed to form either an intra- or inter-connected structure as programmed without any fear of the occurrence of a random crosslink reaction.

Therefore, we thought that the At-ON linkage is an adequate strategy for the purpose. As shown in the model of dual modifications, Oxa and an amine group incorporated in the same DNA strands were employed as upstream and downstream DNA fragments to check the applicability (Fig. 3C; Supplementary Fig. S9A). The model of dual modifications produced the crosslinked strand via the proximity-induced linkage between Oxa (at the 3′ end) and an internal amine group only when two identical reactive strands were recruited in one template strand. In the absence of the template, less than ∼1% of non-specific self-linkage occurred after 48 h incubation at 37°C even though both reaction groups co-exist within the same DNA strand (Supplementary Fig. S9B).

The sequential chain ligation principle introduced in Fig. 4 finds its structural realization in the assembly of T-motif tiles. Because each reactive tile strand carries both an Oxa and an amine group as a coupling pair, every new tile recruited to the growing assembly brings a fresh set of reactive termini, allowing covalent At-ON linkages to propagate across multiple tile–tile junctions without the synthetic constraint of preparing separately functionalized strands for each connection site. This is in direct contrast to DBCO–azide click chemistry, where chain propagation is inherently limited by the requirement for alternating donor and acceptor strands.

**Figure 6.**
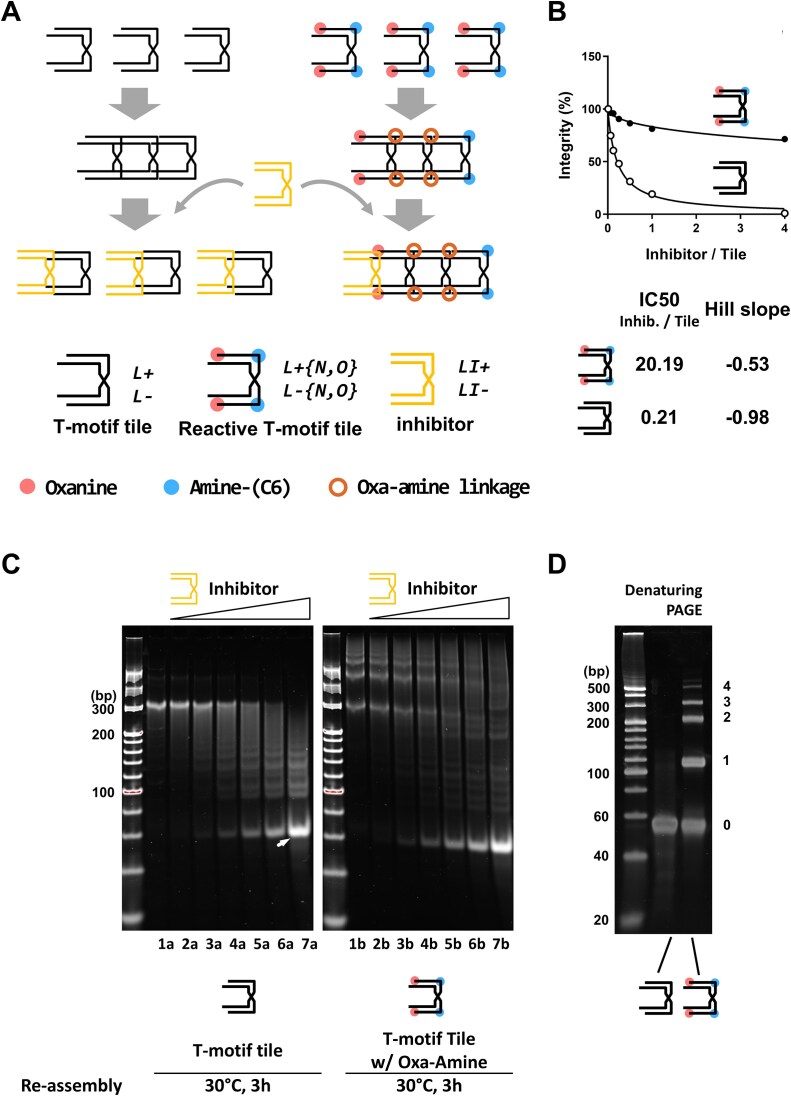
The effect of Oxa–amine linkages on T-motif tiles with the coupling-pair-on-a-strand. (**A**) The assembly of T-motif tiles that generates multimeric structures and its further incubation with or without the inhibitor tiles. T-motif tiles modified with both an internal amine and the 3′ end of Oxa would be crosslinked to each other through the assembly process. (**B, C**) The integrity of the assembled structure was evaluated by measuring the signal intensity observed above the marker standard of 300 bp depending on the inhibitor concentration from the results of native PAGE (6%) stained with SYBR Gold. The inhibitor (white arrow) was applied to the structure, followed by additional incubation. (**D**) The separation result from denaturing PAGE (6%) reveals that the reactive T-motif tiles are inter-connected. The observable chain linkages occurred up to four times.

Next, we employed the At-ON linkage for the multimerization of T-motif tiles based on the results on the junction design of a T-motif (#5 with a 6 bp loop) and in the model of dual modifications (#6). The T-motif tile monomer consists of a dimeric form of T-motifs, in which the assembling pairs that are guiding the overhang and binding loop domain are located at both ends of the tile, which allows tile–tile self-assembly, forming a repetitive structure (Fig. 6A). In our self-assembling system, a non-reactive multimeric structure was formed up to 300 bp based on the DNA size marker, as observed in the native PAGE. Notably, in the case of crosslinkable multimerization (using reactive T-motif tiles with Oxa and amine modification), the size of the multimers was shown to be more elongated than the non-reactive tile (Fig. 6C; lanes 1a and 1b). It can be inferred that the involvement of the Oxa–amine linkage with the formation or during the assembly of DNA architecture gives several irreversible connections to the self-assembled DNA structures, and the resulting structural rigid stability can help the DNA assembly to undergo additional elongation.

To identify the effect of Oxa–amine linkages for the rigid stability of multimerized T-motif tiles, we added inhibitor tiles to two kinds of the assembled DNA architecture product: (i) one architecture consisting of normal T-motif tiles and (ii) the other of reactive T-motif tiles (modified with Oxa and amine). Since the inhibitor tiles have no template overhang, it can only intercept other overhang regions from already-made T-motif multimers. As expected, the multimers of normal T-motif tiles rapidly dissociated as the concentration of initiator tiles increased, revealing the standard inhibitory Hill slope. However, the multimer of reactive T-motif tiles with Oxa–amine linkage could resist treatment with the inhibitor more than that of non-reactive tiles (Fig. 6B, C). When equal amounts of inhibitors were added to the tiles, the degree of preservation was 81.2% for the reactive T-motif tile-based DNA structure, but 19.2% for the normal tile-based DNA structure, compared with the initial amount. Moreover, the ratio of the half-maximal inhibitory ratio of inhibitors per T-motif tile was > 20 (estimated by curve extrapolation) for the Oxa–amine linkage-based DNA structure, which was substantially higher than when normal T-motif tiles were used. Notably, multimerization integrity, defined as the fraction of material migrating above the 300 bp threshold, can remain high even when per-junction linkage efficiency does not individually exceed 50%, because each reactive tile carries two At-ON linkage sites, and partial covalent bond formation across even a subset of junctions collectively sustains the chain-extended structure. The separation result from denaturing PAGE reveals that the reactive T-motif tiles have been inter-connected by up to four tiles sequentially (Fig. 6D).

**Figure 7.**
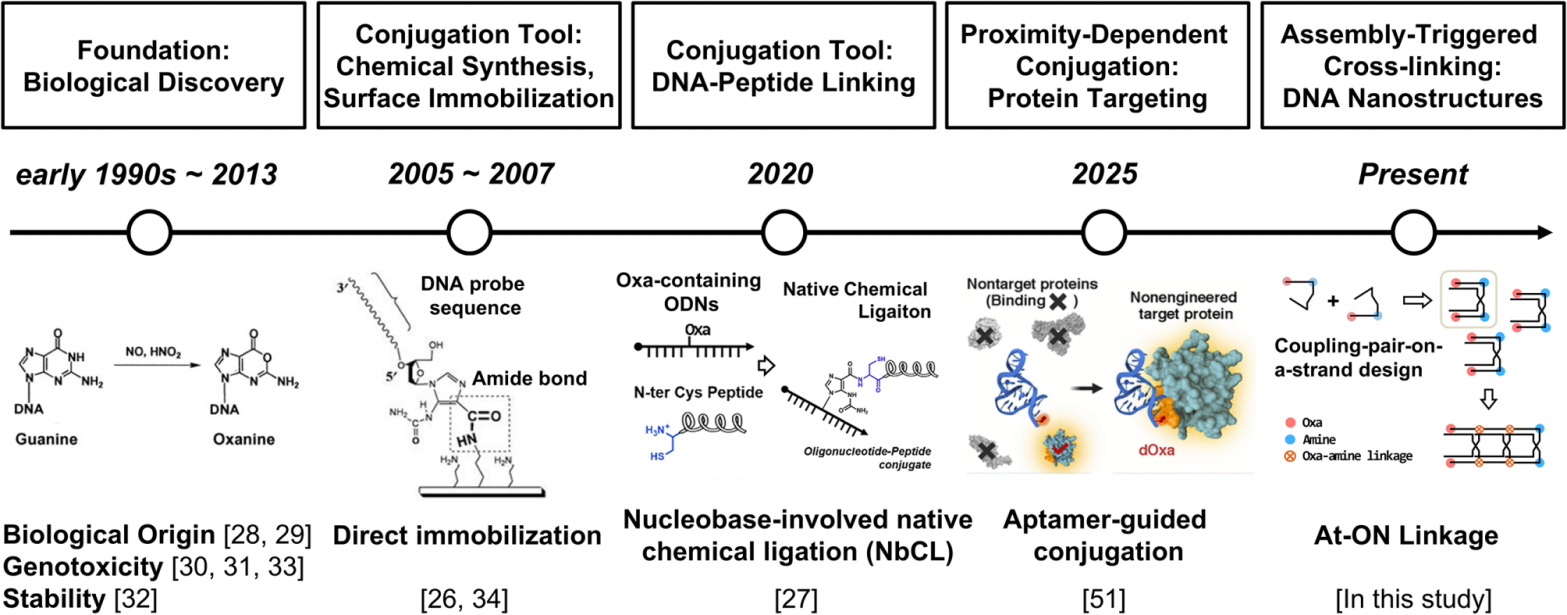
Timeline of oxanine-mediated conjugation strategies leading to the assembly-triggered DNA crosslinking system presented herein.

This study show that the At-ON linkage works across DNA assemblies of different scales, from individual junction motifs to multimerized tile assemblies. Also, the At-ON linkage remained strongly regulated by the presence of atemplate (Figs 2B and 3A, B ), minimizing background (unwanted byproducts) while preserving selective covalent capture. The results expand the possibility of using Oxa-based conjugation for stable and programmable DNA assembly by establishing a heterobifunctional reactive building block, coupling-pair-on-a-strand. Earlier publications [26, 27] reported Oxa-based crosslinking with amine-functionalized small molecules and peptides in non-templated, diffusion-controlled reactions, and a recent aptamer-guided conjugation study [51] applied Oxa chemistry to selective protein conjugation (Fig. 7). In contrast, the present study uniquely harnesses Oxa’s reactivity to construct an DNA assembly-triggered inter-strand crosslinking system in which covalent bond formation is suppressed until DNA hybridization brings reactive partners into proximity. Moreover, by co-incorporating both Oxa and amine modifications on the same DNA strand, which constitutes a coupling-pair-on-a-strand, we further demonstrate that sequential chain ligation across inter-connected DNA nanostructures becomes possible, a design concept enabled by the unique nature of Oxa reactivity. This assembly-triggered crosslinking strategy has broad potential for programmable DNA applications, including stabilization of DNA origami scaffolds, reinforcement of DNA hydrogels, covalent fixation of DNA–nanoparticle conjugates, and topological trapping of interlocked architectures such as catenanes and rotaxanes.

## Conclusion

This study focused on the use of Oxa as an assembly-dependent crosslinker for stable and programmable formation of DNA nanostructures, exploiting its proximity-dependent reactivity to form covalent bonds with amine-modified partner strands upon DNA self-assembly. Oxa, a guanine analog with a unique reactivity toward primary amine groups, is stably maintained in aqueous solution and can be incorporated into DNA strands enzymatically or chemically, and subsequently employed as a crosslinker that reacts with proximal amine groups under catalyst-free conditions. We applied the molecular guiding principle with the kinetically inert and proximity-dependent electrophilicity of Oxa to propose the At-ON (assembly-triggered Oxa–amine) linkage system, which is based on the proximity-induced crosslink formation between Oxa and an amine group within the localized DNA strands. Through various structural DNA motifs including duplex, Y-shapes, and T-motif tiles, the At-ON linkages between Oxa-modified strands and amine-modified strands have been verified with quite low backgrounds. The inverse relationship between At-ON linkage efficiency and reaction temperature in T-motif junctions further confirmed that covalent bond formation is kinetically coupled to the self-assembly process rather than occurring independently.

A functionally distinct feature is the coupling-pair-on-a-strand design, in which both Oxa and amine reside on the same strand and remain unreacted until template strand recruitment. This enables sequential chain ligation that propagates covalent linkages in a self-extending fashion, yielding higher-order concatenated architectures, which are not accessible in DBCO–azide click chemistry. These properties make the At-ON system applicable to the fields employing programmable DNA assembly, including selective DNA glues, hydrogels, and DNA-mediated particle assembly, where irreversible crosslinks enhance structural stability. Current crosslinking yields range from ∼55% to ∼87% depending on the structural motif, with the primary constraint being the conformational rigidity of the C6 amine linker in internal backbone modifications. Product heterogeneity in multi-site crosslinking scenarios must be resolved before application to homogeneous structures such as DNA origami. Future work will target the design of amine linker spacers for non-duplex motifs and site-specific internal Oxa incorporation to improve the crosslinking yield.

## Supplementary Material

gkag638_Supplemental_File

## Data Availability

The data underlying this article are available in the article and in its online supplementary data.
