# Supplementary material for: Stable and programmable formation of DNA nanostructures via inter-strand crosslinking by assembly-triggered oxanine and amine linkage
Source: Nucleic Acids Res. 2026 Jun 22;54(12):gkag638. doi: 10.1093/nar/gkag638 (PMC13284720; doi:10.1093/nar/gkag638)
Supplement: gkag638_Supplemental_File [file gkag638_supplemental_file.docx]

**Supplementary materials**

**Stable and programmable formation of DNA nanostructures via inter-strand crosslinking by assembly-triggered oxanine and amine linkage**

Jae Eon Lee^1,†^, Eui Kyoung Jang^1,†^, Ryeo Gang Son^1^, Jeehyun Park^1^, and Seung Pil Pack^1,*^

^1^ Department of Biotechnology and Bioinformatics, Korea University, Sejong-Ro 2511, Sejong, 30019, Republic of Korea
^†^ These authors contributed equally to this work.

Tel: +82-(44)-860-1419; Email: spack@korea.ac.kr (for SP Pack)

**Table of Contents**

Figure S1-----------------------------------------------------------------------------------------------------------2

Figure S2-----------------------------------------------------------------------------------------------------------3

Figure S3-----------------------------------------------------------------------------------------------------------4

Figure S4-----------------------------------------------------------------------------------------------------------5

Figure S5-----------------------------------------------------------------------------------------------------------6

Figure S6-----------------------------------------------------------------------------------------------------------7

Figure S7-----------------------------------------------------------------------------------------------------------8

Figure S8-----------------------------------------------------------------------------------------------------------9

Figure S9----------------------------------------------------------------------------------------------------------10

Figure S10---------------------------------------------------------------------------------------------------------11

Numerical simulation-------------------------------------------------------------------------------------------12

Table S1----------------------------------------------------------------------------------------------------------16

Table S2----------------------------------------------------------------------------------------------------------18

Table S3----------------------------------------------------------------------------------------------------------19

References-------------------------------------------------------------------------------------------------------20


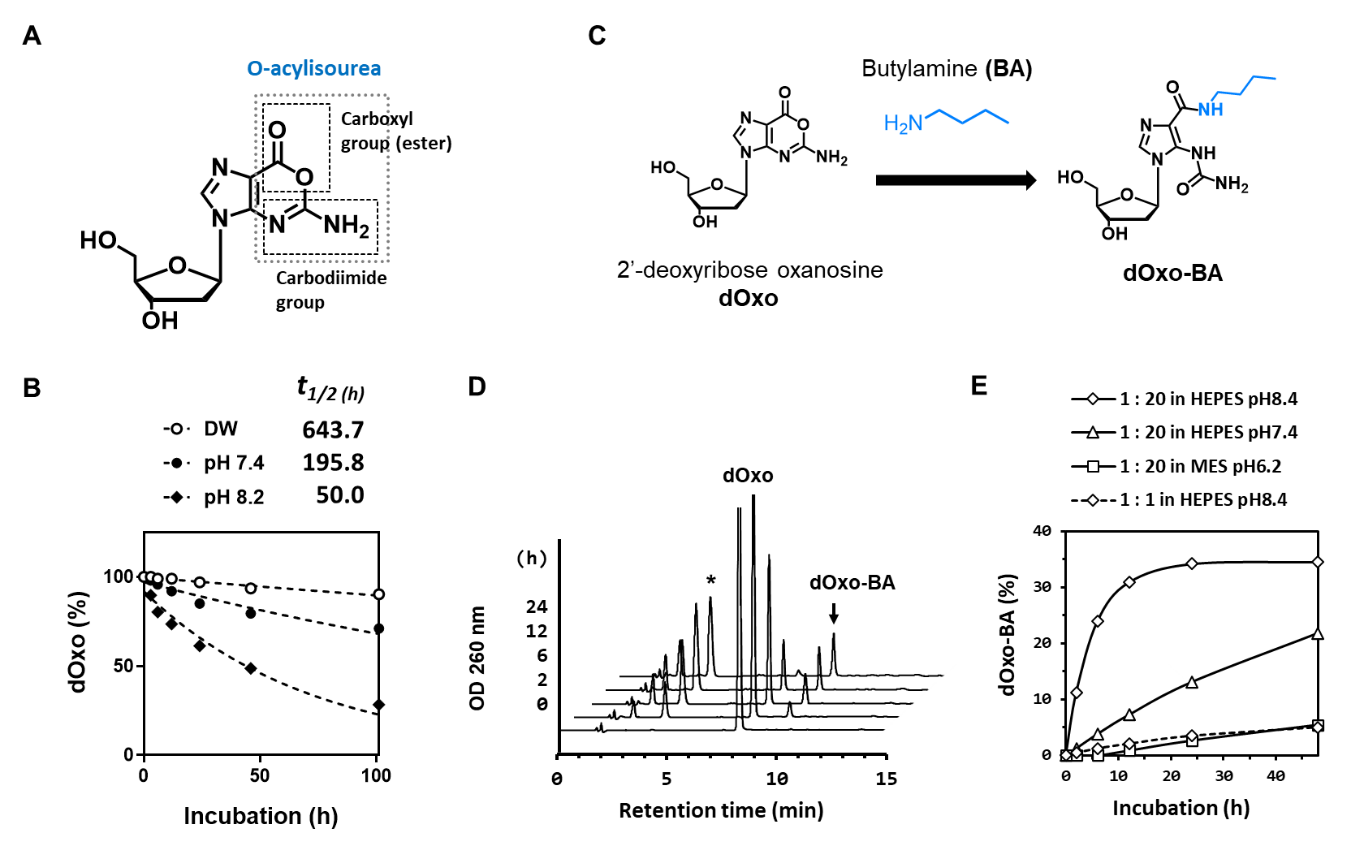


**Figure S1. The monomer reaction between Oxa-containing dOxo and butylamine as a monoamine.** A) The compound of deoxyribose form (dOxo) of oxanine (Oxa). B) The residual dOxo was measured after incubating 3 mM of dOxo in DW, pH 7.4 and pH 8.2 at 24 °C. ***t_1/2_*** refers to the half-life of dOxo, derived from the exponential decay curve. C) The reaction of butylamine to the carbodiimide-activated carboxylate form of Oxa results in a covalent bond-formed complex, dOxo-BA. D) The reaction product that was generated by incubation of 1 mM of dOxo with 20 mM of BA in 300 mM of HEPES buffer pH 8.4 at 24 °C was analyzed by the HPLC system. A star (*) refers to dXao, which is an inactive compound converted from dOxo when it is exposed in basic condition [1]. E) The production of dOxo-BA at different reaction pH was analyzed over time, comparing the use of 1 mM and 20 mM of BA was compared.


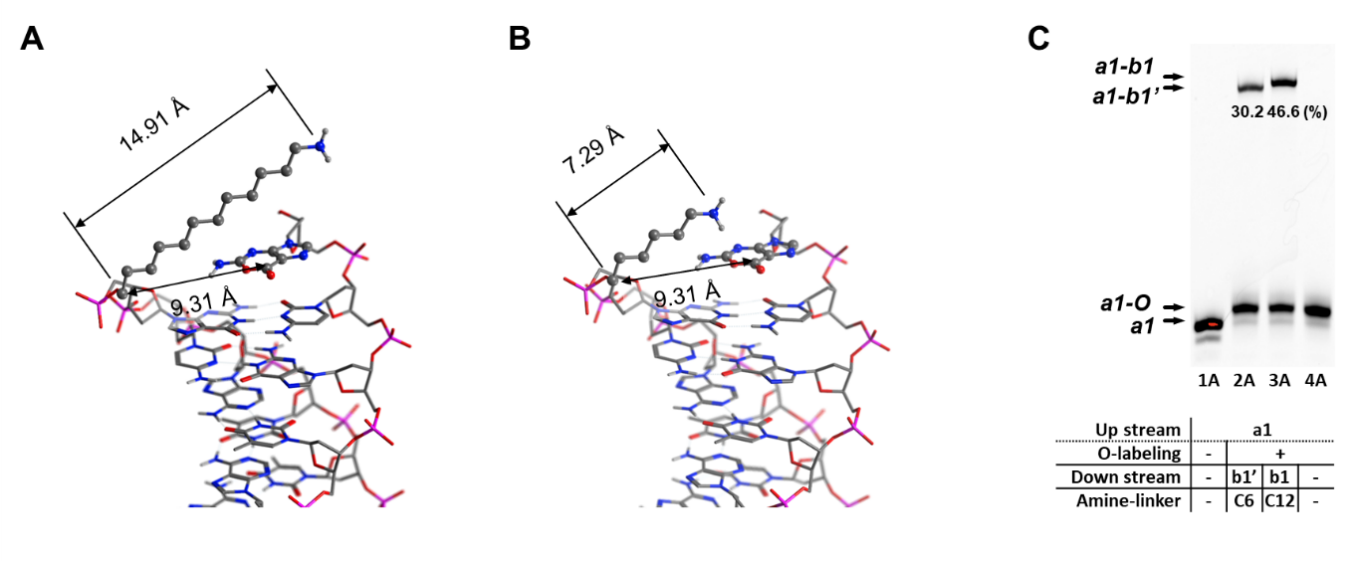


**Figure S2. Effect of two types of amine linkers.** A) The length of the stretched C12 linker of amine (14.91 Å) is longer than the length between C12 of linker and C1 of Oxa (9.31 Å). B) The length of the stretched C6 linker of amine (7.29 Å) is shorter than the length between C6 of linker and C1 of Oxa (9.31 Å). C) In the type **#1** of Oxa-amine linkage, C12 exhibits 1.5 times more reactivity than C6.


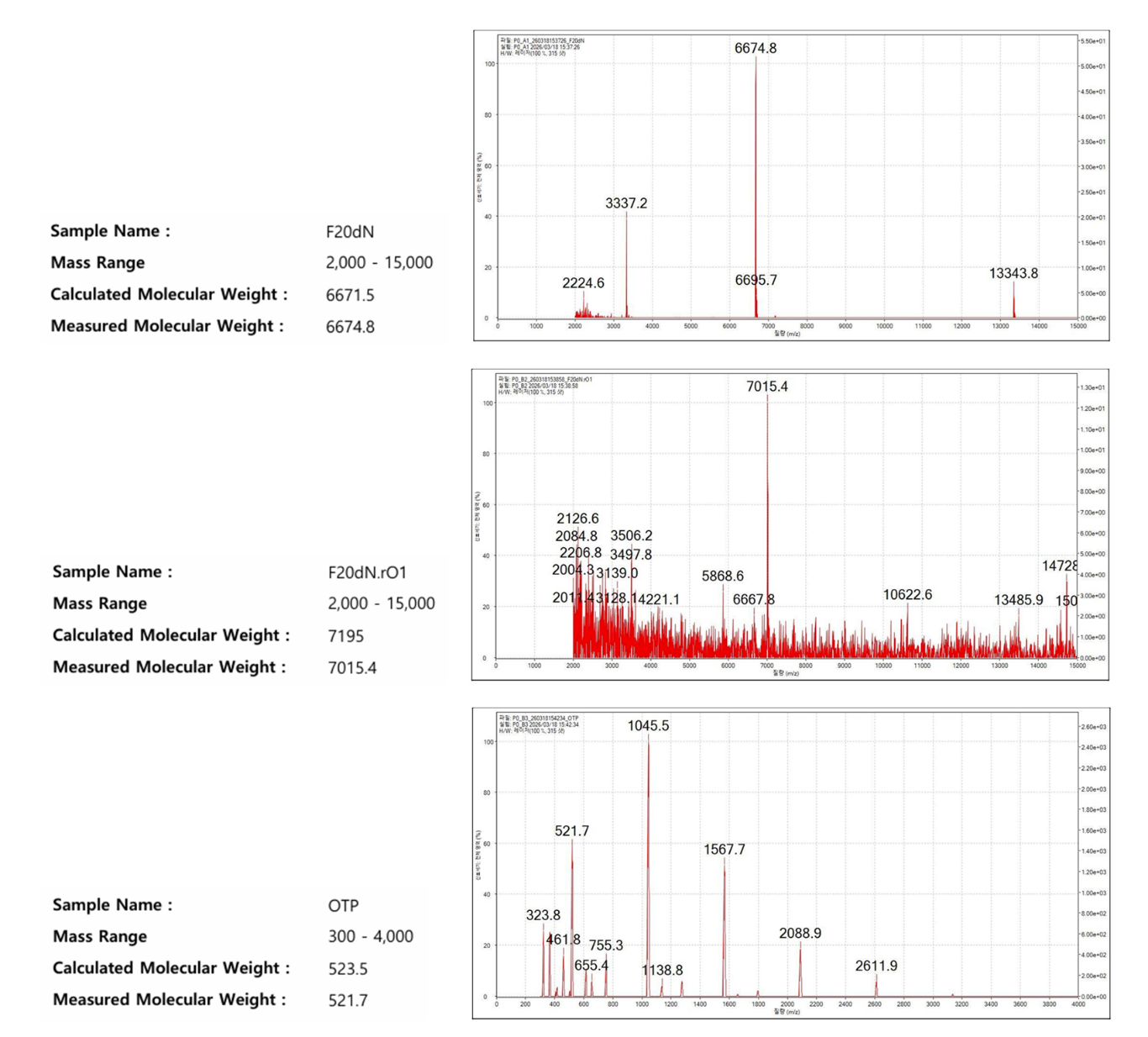


**Figure S3. MALDI-TOF MS analysis confirming single incorporation of OTP into F20dN by TdT-mediated 3′-extension.** Mass spectrometric verification of OTP, the starting oligonucleotide (F20dN), and its TdT-extended product (F20dN.rO1). (Top) The measured molecular weight of F20dN (6674.8 Da) is in close agreement with the calculated value (6671.5 Da), confirming the identity of the substrate strand. (Middle) Following TdT reaction with OTP, the product F20dN.rO1 shows a measured molecular weight of 7015.4 Da. The observed mass increment (Δm = +340.6 Da relative to F20dN) corresponds to the addition of a single Oxa nucleoside monophosphate residue, consistent with one-nucleotide extension and concomitant release of pyrophosphate (PPi, ~178 Da) during TdT-catalyzed incorporation.


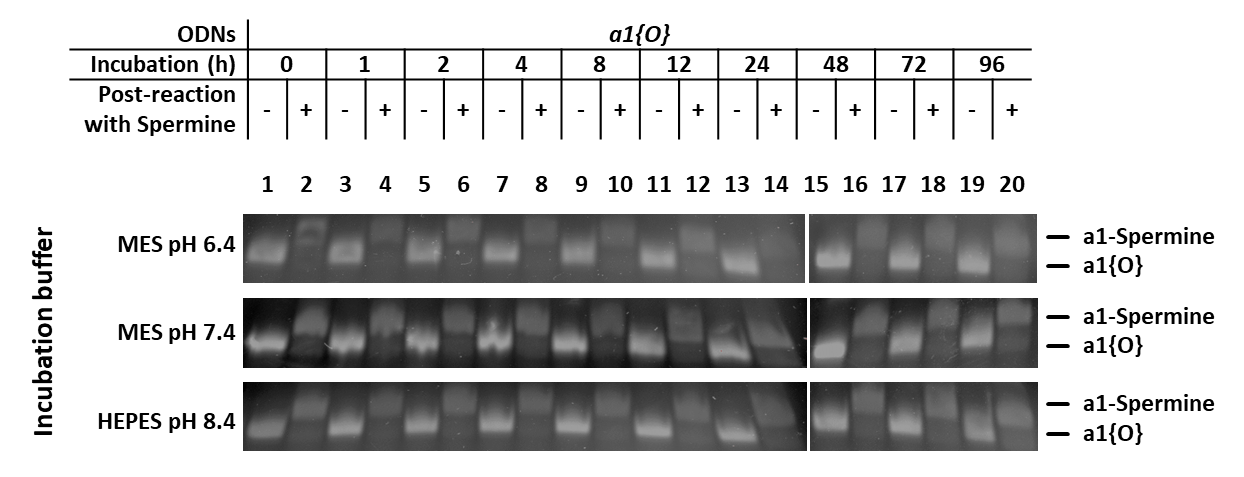


**Figure S4. The sustainability of Oxa reactivity when incorporated at the end of the DNA strand.** A) To determine the loss of reactivity by hydrolysis, Oxa-labeled DNA strand (***a1{O}***) was incubated under varying pH conditions, then the residual reactivity of Oxa was assessed via its reaction with excess amine molecules (spermine). Specifically, 0.5 μM Oxa-labeled DNA strand was incubated in 5 mM buffers, followed by treatment with 33000 times excess spermine. B) The residual reactivity was calculated from the PAGE result (C), where residual reactivity was measured by the ratio of FI_products / (FI_products + FI_remains) and normalized by dividing the residual reactivity at each time point by the initial value (t0).


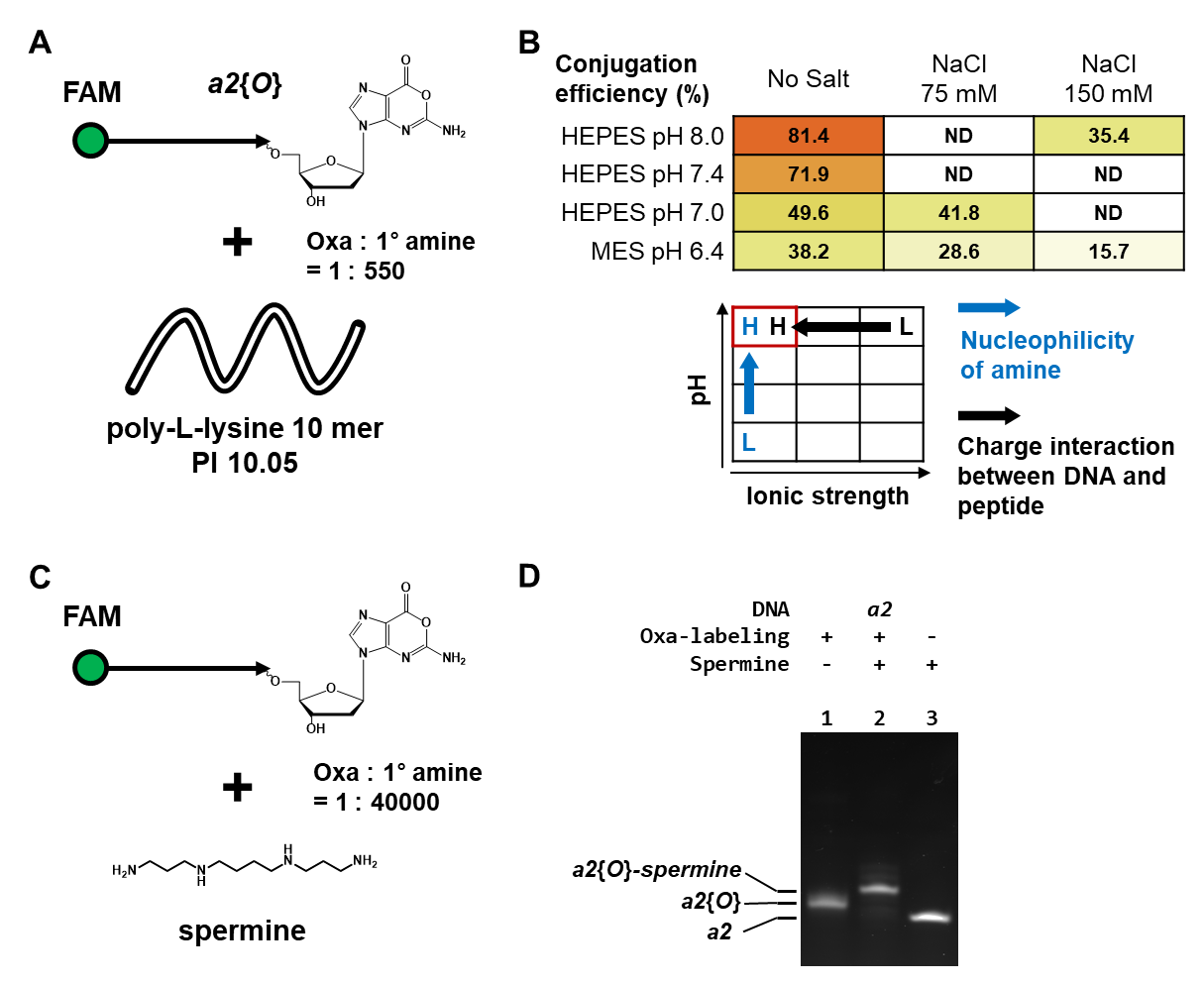


**Figure S5. The effect of macromolecular interaction for the Oxa-amine reaction.** A) The charged interaction between Oxa-modified DNA (***a2*{*O*}**) and cationic peptide (poly-_L_-lysine; 10 mer lysine) was evaluated as a model of macromolecular interaction. B) The efficiency of the reaction was directly proportional to the extent of charge interaction between the lysine residues and the DNA backbone. The interaction is regulated by the ionic strength in solution. 4 μM of Oxa-modified DNA reacted with 200 μM of peptides in the reaction buffers with varying pH and NaCl concentrations. These results were measured after separation on denaturing PAGE. ND means no data. C) The reaction of Oxa-modified DNA with excessive amounts of spermine was conducted with the condition at 37 °C for 1 hour in HEPES pH 8.4. D) The result of denaturing PAGE reveals that most of the Oxa-modified DNA reacted with spermine.


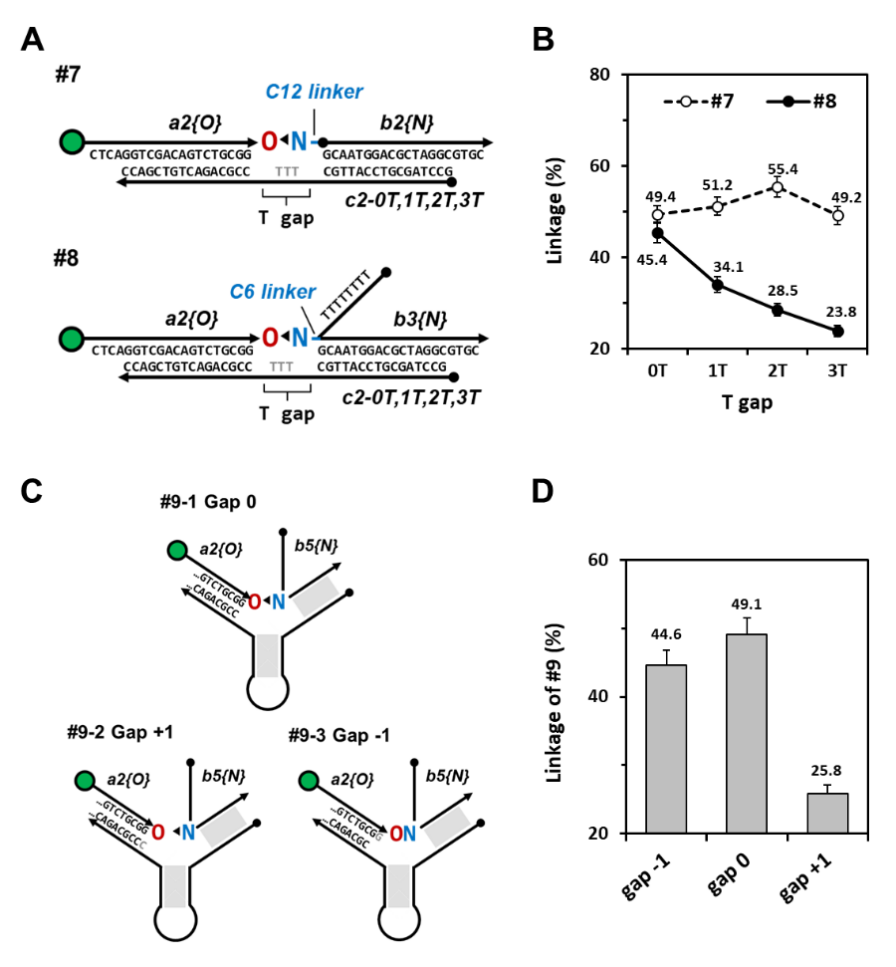


**Figure S6. The distance-dependent reactivity in linear- and three-way junctional motifs.** A) Two linear shapes of Oxa-amine linkages including amine’s linkers for C6 and C12 in each have been evaluated for several thymine gaps. B) The length of linkers determines the most efficient gap length, with the C12 linker (**#7**) being suitable for two thymine gaps and the C6 linker (**#8**) being suitable for no gap. C) The optimal gap for ODLs in DNA three-way junctions has also been considered by using template-mediated annealing. There are three variants in which a single base moves forward (**#9-3**) or backward (**#9-2**) from the no-gap position (**#9-1**) toward the amine functional group on ***b5***. D) The linkage efficiency shows the optimal length of the gap for the interstrand amine (with C6 linker) modification in the DNA junctional motif. The error indicates the range of standard deviation (N=3).


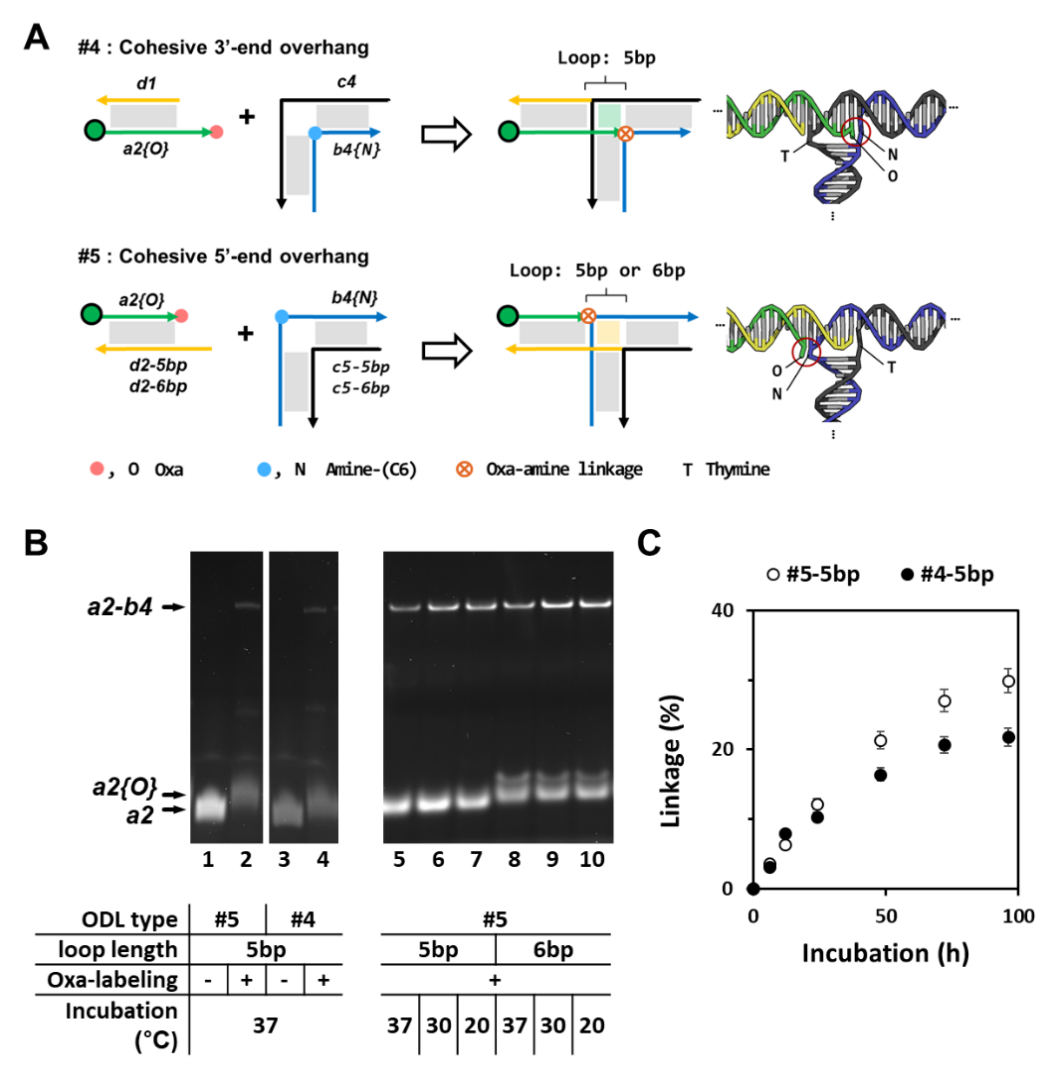


**Figure S7. The design of assembly-triggered Oxa-amine linkages in T-motif.** A) There are two T-motif Oxa-amine linkage subtypes based on the position of Oxa, which are overhanging Oxa (**#4**) and recessive-end Oxa (**#5**). Oxa at **#4** makes a crosslink with the amine at the inner strand (***b4*{*N*}**) by directly annealing with the loop strand (***c4***). Otherwise, Oxa at **#5** bonds with the loop strand by assist of the template strand (***d2***). B) The linkages (***a2-b4***) for both T-motifs (**#4** and **#5**) are only observed in Oxa-labeled sequences, and the optimal reaction temperature was identified. C) The reaction products from **#4** and **#5** were measured the linkage efficiency for incubation time at 20 ℃.


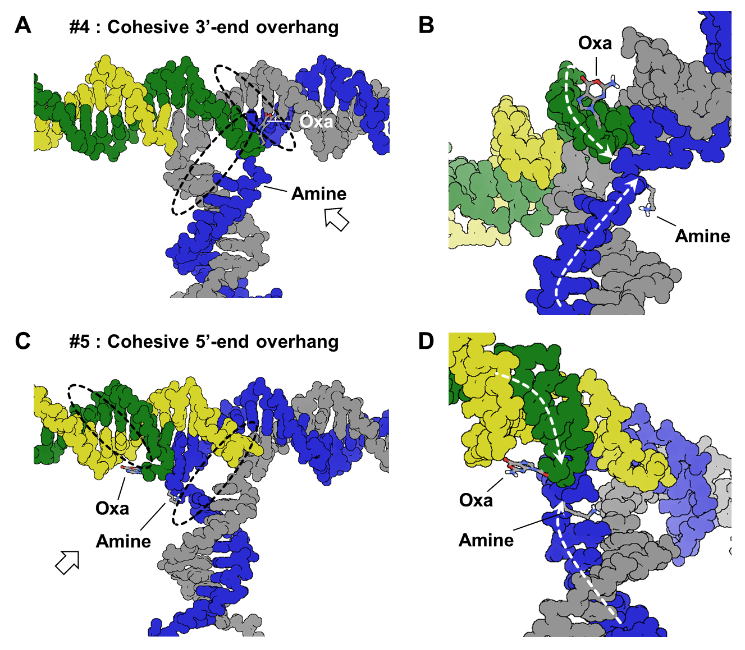


**Figure S8. Two T-motif Oxa-amine linkages and the differences in structure surrounding the reaction groups.** A) The 3′-end overhang T-motif (**#4**) is configured by the interaction of the guiding sequence in the Oxa-modified strand (green) and the loop strand. B) Oxa is situated within a cavity in one of the major grooves (dotted circle) in structure **#4**. However, the movement of the amine group appears to be impeded by the phosphate backbone of the strand that crosses the cavity. C) The 5′-end overhang T-motif (**#5**) is formed through the interaction between the 5′-end region of the guiding strand (highlighted in yellow) and the loop strand that contains the amine group. D) In structure #5, two reactive groups are positioned at the junction of two major grooves. This model was designed using oxView [2] and visualized using ChimeraX [3] with energy minimization step.


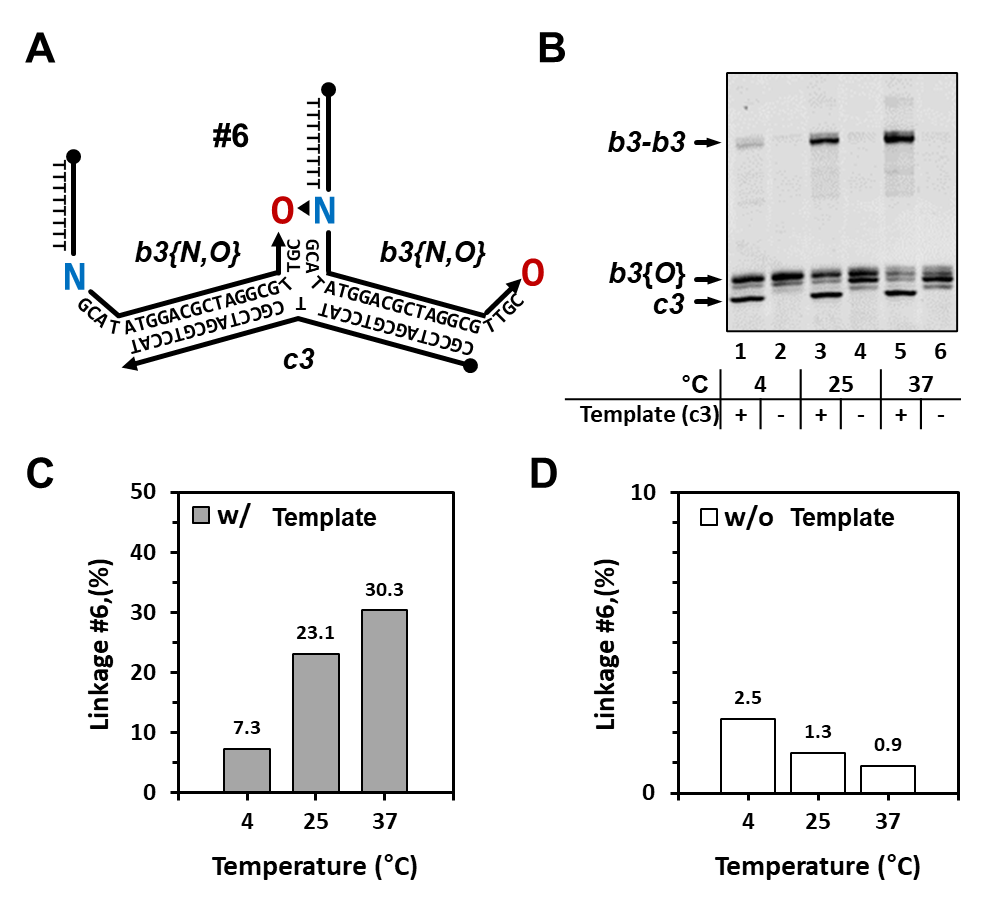


**Figure S9. Assembly-triggered Oxa-amine linkage when the pair of coupling groups (Oxa and amine) are included within one DNA strand.** A) Design of coupling pair within one strand, the model of dual modifications (**#6**). When two identical strands modified with a coupling pair (***b3*{*N,O*}**) are recruited in a specific template (***c3***), covalent bonding takes place. B) The Oxa-amine linkage results on denaturing PAGE indicate the reaction is triggered by the association of template strand, depending on the reaction temperature. C, D) The plot of template-dependent reaction for **#6** was quantified from B). Higher reaction temperatures lead to an increased formation of linkages in these templated reactions. However, at lower temperatures, there is a higher occurrence of auto self-reactions, suggesting that the unstable interaction involving three bases at the stem region, without a template strand, were stabilized at the lower temperature.


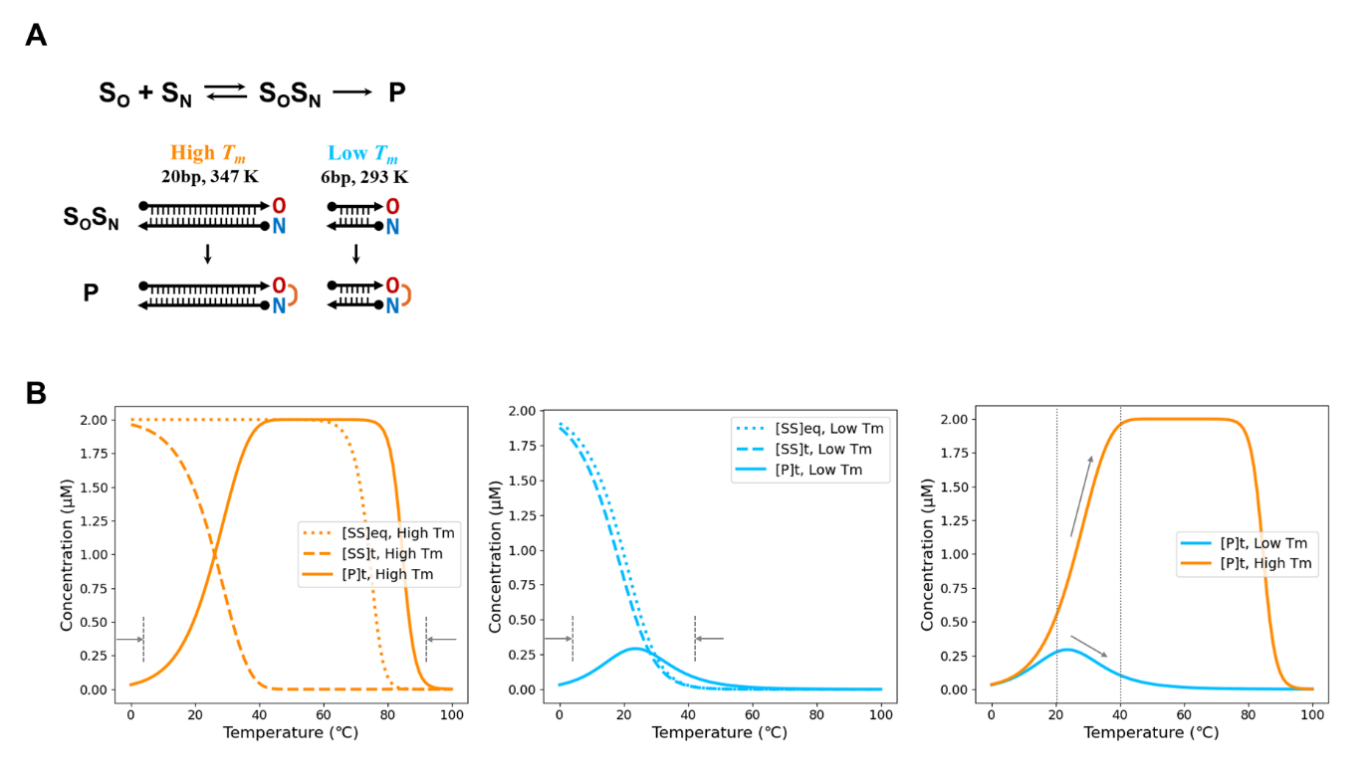


**Figure S10. Numerical simulation of the trends in assembly-specific linkage production depend on reaction temperature.** A) Oxa-amine reactions in DNA strands have been described using two-state models with high and low melting temperatures. B) Numerical simulations show the concentration change of the reaction products across a range of reaction temperatures, indicating different melting temperature dependencies for both models.

**Numerical simulation**

The simplified model of assembly-triggered Oxa-amine linkage

To demonstrate the trends in assembly-specific linkage production depending on reaction temperature, the Oxa-amine reaction mediated by DNA complementary binding was represented by the following model:

$$\boldsymbol{A}\boldsymbol{+}\boldsymbol{B} \boldsymbol{\leftrightarrow}\boldsymbol{AB} \boldsymbol{\to}\boldsymbol{P}$$

The rate of change of the concentration of each reactant in the model can be expressed by the following differential equations:

$\frac{d\left[ A \right]}{dt}=-k_{f}\left[ A \right]\left[ B \right]+k_{r}\left[ AB \right]$

$\frac{d\left[ B \right]}{dt}=-k_{f}\left[ A \right]\left[ B \right]+k_{r}\left[ AB \right]$

$\frac{d\left[ AB \right]}{dt}=k_{f}\left[ A \right]\left[ B \right]-k_{r}\left[ AB \right]-k_{p}\left[ AB \right]$

$\frac{d\left[ P \right]}{dt}=k_{p}\left[ AB \right]$

The aim of numerical simulation is to conceptually visualize the concentration changes of each reactant concerning reaction temperatures and melting temperatures. Therefore, we calculated the rate constants by applying the Arrhenius equation with predetermined constants.

$k=A\cdot\exp\left( \frac{-E_{a}}{RT} \right)$ (equation 1)

The activation energy (*E_a_*) and the pre-exponential factor (*A*) for the duplex forming rate constant (*k_f_*) were obtained from the thermodynamic parameters reported by the Holyst group [5]. The dissociation rate constant (*k_r_*) was determined using the definition of *K_d_* (= *k_r_* / *k_f_*). Under saturated hybridization conditions ([A₀] ≫ *K_d_*), [AB] ≈ [A₀] − [P], and the reaction follows first-order kinetics: d[P]/dt = *k_p_*([A₀] − [P]). *k_obs_* were extracted by fitting experimental data to this integrated rate law, where *k_obs_* can be regarded as the effective *k_p_* under the given conditions.

The relationship between *T*, *T_m_* and *K_d_*

Expressing the Van't Hoff equation in terms of the dissociation equilibrium constant gives the following:

$\ln K_{d}=\frac{\Delta H}{RT}-\frac{\Delta S}{R}$ (equation 2)

Here, *R* is the gas constant, and *T* is the reaction temperature. At the melting temperature *T_m_*​, the dissociation constant *K_d_​* can be expressed as:

$K_{d}\left( T_{m} \right)=\frac{\left[ A \right]\left[ B \right]}{\left[ AB \right]}=\frac{\left[ A_{0} \right]}{2}$ (equation 3)

Taking the natural logarithm of both sides gives:

$\ln K_{d}\left( T_{m} \right)=\frac{\Delta H}{RT_{m}}-\frac{\Delta S}{R}=\ln\left( \frac{\left[ A_{0} \right]}{2} \right)$ (equation 4)

Solving for the entropy change Δ*S*:

$\Delta S=R\left( \frac{\Delta H}{RT_{m}}-\ln\left( \frac{\left[ A_{0} \right]}{2} \right) \right)$ (equation 5)

Assuming entropy and enthalpy are constant with respect to temperature, and substituting Equation 5 into Equation 2, we obtain the expression for *K_d_*​ in terms of temperatures *T* and *T_m_*​:

$K_{d}=\frac{\left[ A_{0} \right]}{2}\cdot e^{\left( \frac{\Delta H}{R}\left( \frac{1}{T}-\frac{1}{T_{m}} \right) \right)}$ (equation 6)

Equilibrium concentration of AB complex

When the initial concentrations of A and B are equal, assuming [AB]_eq_ = x, then [A]_eq_ = [B]_eq_ = [A_0_] – x. Hence, the dissociation constant *K_d_*​ can be expressed as follows:

$K_{d}=\frac{\left( \left[ A_{0} \right]-x \right)^{2}}{x}$ (equation 7)

This simplifies to the quadratic equation:

$x^{2}-\left( 2\left[ A_{0} \right]+K_{d} \right)x+\left[ A_{0} \right]^{2}=0$ (equation 8)

Therefore, the equilibrium concentration of AB expressed in terms of the equilibrium constant is:

$\left[ AB \right]=\frac{\left( 2\left[ A_{0} \right]+K_{d} \right)-\sqrt{K_{d}^{2}+4K_{d}\left[ A_{0} \right]}}{2}$ (equation 9)

import numpy as np

from scipy.integrate import odeint

import matplotlib.pyplot as plt

# Common parameters

C0 = 2e-6 # Initial concentration [A]0 = [B]0 (M)

T0 = 297 # standard temperature (K) for initial kinetic parameters

R = 8.314  # Gas constant (J/(mol*K))

# The enthalpy (ΔH) and entropy (ΔS) were calculated using NN thermodynamic parameters proposed by Santalucia J Jr (1998).

# Weak binding model (6 bp: GTGTCG)

delta_H_w = -1.8370e+05 # J/mol

delta_S_w = -5.1250e+02 # J/(mol·K)

Tm_w = delta_H_w / (delta_S_w + R * np.log(C0/2))

# Strong binding model (20 bp: TAGCAACCACACGGTAATGC)

delta_H_s = -6.5530e+05 # J/mol

delta_S_s = -1.7712e+03 # J/(mol·K)

Tm_s = delta_H_s / (delta_S_s + R * np.log(C0/2))

Eaf = 6.5e+4 # (J/mol)

Af = 3.4e+15 # (1/(M*s))

# Resolving rate constant (kp) for assembly-triggered Oxa-amine linkages from the result of strong binding model; calulating Ea_p value from kobs of the 20 bp model

kobs_SS = 0.000012 # Observable rate constant (s-1), first order with respect to AB

kf0 = Af * np.exp(-Eaf / (R * T0)) # Arrhenius equation

kp0 = kobs_SS # Since kobs_SS is already in s-1, we can directly use it as kp0

Ap = 1e11 # Pre-exponential factor for kp (1/s). Assumption.

Ea_p = R * T0 * np.log(Ap / kp0) # Activation energy for kp (J/mol)

def calculate_Kd(T, delta_H, Tm):

    Kd = (C0 / 2) * np.exp((delta_H / R) * (1/T - 1/Tm))

    return Kd

def reaction_rates(y, t, kf, kr, kp):

    A, B, AB, P = y

    dA_dt = -kf * A * B + kr * AB

    dB_dt = -kf * A * B + kr * AB

    dAB_dt = kf * A * B - kr * AB - kp * AB

    dP_dt = kp * AB

    return [dA_dt, dB_dt, dAB_dt, dP_dt]

def AB_eq_concentration(T, delta_H, Tm):

    Kd = calculate_Kd(T, delta_H, Tm)

    term1 = np.sqrt(4 * C0 / Kd + 1)

    return (2 * C0 + Kd - Kd * term1) / 2

# Temperature range

temperatures = np.linspace(273.15, 373.15, 100)  # Range from 290K to 360K

t_end = 12 * 3600  # hours in seconds

t = np.linspace(0, t_end, 2) # time frame

# Arrays to store results

AB_eq_weak = []

AB_final_weak = []

P_final_weak = []

AB_eq_strong = []

AB_final_strong = []

P_final_strong = []

for T in temperatures:

    kp = Ap * np.exp(-Ea_p / (R * T))  # AB -> P rate constant (1/s)

    y0 = [C0, C0, 0.0, 0.0]  # Initial concentrations [A]0, [B]0, [AB]0, [P]0

    # Weak binding model

    Kd_w = calculate_Kd(T, delta_H_w, Tm_w)

    kf_w = Af * np.exp(-Eaf / (R * T))  # A + B -> AB rate constant (1/(M*s))

    kr_w = kf_w * Kd_w  # AB -> A + B rate constant (1/s)

    solution_weak, info_w = odeint(reaction_rates, y0, t, args=(kf_w, kr_w, kp), full_output = 1, rtol=1e-7, atol=1e-11)

    AB_final_weak.append(solution_weak[-1, 2] * 1e6)

    P_final_weak.append(solution_weak[-1, 3] * 1e6)

    AB_eq_weak.append(AB_eq_concentration(T, delta_H_w, Tm_w) * 1e6)

    # Strong binding model

    Kd_s = calculate_Kd(T, delta_H_s, Tm_s)

    kf_s = Af * np.exp(-Eaf / (R * T))  # A + B -> AB rate constant (1/(M*s))

    kr_s = kf_s * Kd_s  # AB -> A + B rate constant (1/s)

    solution_strong, info_s = odeint(reaction_rates, y0, t, args=(kf_s, kr_s, kp), full_output = 1, rtol=1e-7, atol=1e-11)

    AB_final_strong.append(solution_strong[-1, 2] * 1e6)

    P_final_strong.append(solution_strong[-1, 3] * 1e6)

    AB_eq_strong.append(AB_eq_concentration(T, delta_H_s, Tm_s) * 1e6)

### Plot results

plt.figure(figsize=(6, 5))

plt.plot(temperatures - 273.15, AB_eq_weak, ':', label='[SS]eq, Low Tm', color='deepskyblue', linewidth=3)

plt.plot(temperatures - 273.15, AB_final_weak, '--', label='[SS]t, Low Tm', color='deepskyblue', linewidth=3)

plt.plot(temperatures - 273.15, P_final_weak, '-', label='[P]t, Low Tm', color='deepskyblue', linewidth=3)

plt.plot(temperatures - 273.15, AB_eq_strong, ':', label='[SS]eq, High Tm', color='darkorange', linewidth=3)

plt.plot(temperatures - 273.15, AB_final_strong, '--', label='[SS]t, High Tm', color='darkorange', linewidth=3)

plt.plot(temperatures - 273.15, P_final_strong, '-', label='[P]t, High Tm', color='darkorange', linewidth=3)

plt.xlabel('Temperature (℃)', fontsize=16)

plt.ylabel('Concentration (μM)', fontsize=16)

# plt.legend(loc='center left', bbox_to_anchor=(0.55, 0.5), fontsize=14)

plt.legend(fontsize=14)

plt.grid(False)

plt.xticks(fontsize=13)

plt.yticks(fontsize=13)

plt.tight_layout()

plt.show()

**Table S1.** Comparison of DNA interstrand connection/crosslinking strategies.

| Method | Catalyst Required | External Activation | Approx. Yield (%) | Background Crosslinking | Applicable DNA Motifs | Coupling-pair-on-a-strand | Key Reference(s) |
| --- | --- | --- | --- | --- | --- | --- | --- |
| Enzymatic Ligation (T4 DNA Ligase) | T4 Ligase  + ATP | ATP | >90% (nicked duplex) | Negligible | Nicked duplexes only; linear products | No | Standard biochemistry |
| CuAAC Click Chemistry | Cu(I) | Cu(I) / reductant | Variable | High (any proximate azide + alkyne) | Broad;  azide + alkyne | No (inter-strand only) | Fantoni et al., Chem. Rev. 2021 |
| SPAAC / DBCO–Azide | None | None | Variable | Moderate (any accessible DBCO + azide) | Broad;  DBCO and azide | No (inter-strand only) | Fantoni et al., Chem. Rev. 2021 |
| Pt(II)–TFO* Click Crosslinking | Cu(I) Pt(II) | Acidic pH (Hoogsteen triplex) | Partial (not fully quantified) | Possible non- specific Pt-DNA adducts | Limited to purine-rich sequences with TFO | No | Hennessy et al., Angew. Chem. 2022 |
| T-T UV Dimerization | None | UV (254, 302 nm) | ~10–36% | High (UV causes global T–T dimerization) | Limited to T–T contact sites in duplexes | No | Basu et al., Biochemistry 2023 and others |
| Psoralen Photo-Crosslinking | None | UV (320–400 nm) | ~60% | High (random intercalation + UV) | Limited  to 5'-TA-3' sequences; risk of off-target adducts | No | Rajendran et al., J. Am. Chem. Soc. 2011 and others |
| Oxa–Amine At-ON Linkage (This study) | **None** | **None** | **~55–87%** **(motif-** **dependent)** | **<1%** **(assembly-** **triggered)** | **Duplex, Y-shape,** **T-motif tiles,** **multimerization;** **broad applicability** | **Yes** **(coupling-** **pair-on-a-strand** **design)** | **This study** |

* TFO: triplex-forming oligonucleotides

**Table S2. Strands for Oxa-amine linkage for DNA interconnection**

|  | **Mod.** | **Seq.** | **mer** |
| --- | --- | --- | --- |
| ***a1*{*O*}** | 5’FAM  3’Oxa | {FAM}TAGCAACCACACGGTAATGC{O} | 20+1 |
| ***a2*{*O*}** | 5’FAM  3’Oxa | {FAM}CTCAGGTCGACAGTCTGCGG{O} | 20+1 |
| ***a2’*{*O*}** | 3’Oxa | CTCAGGTCGACAGTCTGCGG{O} | 20+1 |
| ***a2’*{*D*}** | 3’DBCO | CTCAGGTCGACAGTCTGCGG{DBCO} | 20 |
| ***b1*{*N*}** | 5’Am-C12 | {N}GCATTACCGTGTGGTTGCTAGGCGTGC | 27 |
| ***b1’*{*N*}** | 5’Am-C6 | {N}GCATTACCGTGTGGTTGCTAGGCGTGC | 27 |
| ***b1’*{*N3*}** | 5’Azide | {N3}GCATTACCGTGTGGTTGCTAGGCGTGC | 27 |
| ***b1’*{*N*,O}** | 5’Am-C12  3’Oxa | {N}GCATTACCGTGTGGTTGCTAGGCGTGC{O} | 27+1 |
| ***b2*{*N*}** | 5’Am-C12 | {N}GCAATGGACGCTAGGCGTGC | 20 |
| ***b3*{*N*}** | Int Am-C6 | TTTTTTTT {N} GCA ATGGACGCTAGGCG TGC | 28 |
| ***b3*{*N,O*}** | Int Am-C6  3’Oxa | TTTTTTTT {N} GCA ATGGACGCTAGGCG TGC{O} | 28+1 |
| ***b4*{*N*}** | Int Am-C6 | CGGGAATGAGCCTTCC {N} GCAACACACGCCTTCGCCAAGAGATGC | 43 |
| ***b5*{*N*}** | Int Am-C6 | TATGAAAGGCAGCGC {N}  GCATCTCTTGGCGTCGTCTTCTGGTGC | 42 |
| ***c1*** | - | CGCCTAGCGTCCATTTACCGTGTGGTTG | 28 |
| ***c2-0T*** | - | GCCTAGCGTCCATTGC CCGCAGACTGTCGACC | 32 |
| ***c2-1T*** | - | GCCTAGCGTCCATTGC T CCGCAGACTGTCGACC | 33 |
| ***c2-2T*** | - | GCCTAGCGTCCATTGC TT CCGCAGACTGTCGACC | 34 |
| ***c2-3T*** | - | GCCTAGCGTCCATTGC TTT CCGCAGACTGTCGACC | 35 |
| ***c3*** | - | CGCCTAGCGTCCAT T CGCCTAGCGTCCAT | 29 |
| ***3w*** | - | GACGCCAAGAGATGC CCACATGCTTTTGCATGTGG CCGCAGACTGTCGAC | 50 |
| ***3w+*** | - | GACGCCAAGAGATGC CCACATGCTTTTGCATGTGG CCCGCAGACTGTCGA | 50 |
| ***3w-*** | - | GACGCCAAGAGATGC CCACATGCTTTTGCATGTGG CGCAGACTGTCGACC | 50 |
| ***4w*** | - | GACGACGCCAAGAGA CCACATGCTTTTGCATGTGG TTACCGTGTGGTTGC | 50 |
| ***c4*** | - | AGGCGTGTGTTGCCCGCATGGAAGGCTCATTCCCG | 35 |
| ***c5-5bp*** | - | GCATCTCTTGGCGAAGGCGTGTTGGAAGGCTCATTCCCG | 39 |
| ***c5-6bp*** | - | GCATCTCTTGGCGAAGGCGTGAGGAAGGCTCATTCCCG | 38 |
| ***c6*** | - | CCTAGCAACCACACGGTAATGC TT CCGCAGACTGTCGACC | 40 |
| ***c7*** |  | CACACGGTAATGC TT GCACGCCTAGCAAC | 29 |
| ***d1*** | - | GACTGTCGACCTGAG | 15 |
| ***d2-5bp*** | - | GTTGCCCGCAGACTGTCGACCTGAG | 25 |
| ***d2-6bp*** | - | TGTTGCCCGCAGACTGTCGACCTGAG | 26 |
| ***L+*** | - | GTGTCGAGTAACACGAAGCCA ACCTCTAAATCTCCA CGACACTCAGCATCCGATTTG | 57 |
| ***L-*** | - | TGCAGTCAAATCGGATGCTGA TGGAGATTTAGAGGT ACTGCATGGCTTCGTGTTACT | 57 |
| ***LI+*** | - | AGTAACACGAAGCCA ACCTCTAAATCTCCA CGACACTCAGCATCCGATTTG | 51 |
| ***LI-*** | - | CAAATCGGATGCTGA TGGAGATTTAGAGGT ACTGCATGGCTTCGTGTTACT | 51 |
| ***L+*{*N,O*}** | 3’Oxa  int Am | GTGTCGAGTAACACGAAGCCA ACCTCTAAATCTCC{N} CGACACTCAGCATCCGATTTG{O} | 56+1 |
| ***L-*{*N,O*}** | 3’Oxa  int Am | TGCAGTCAAATCGGATGCTGA AGGAGATTTAGAGG{N} ACTGCATGGCTTCGTGTTACT{O} | 56+1 |

**Table S3.** **The effect of Oxa-amine linkages in various assembled structures.**

| **ID** | **Conformation** | **Location of the coupling pairs** | **Amine linker** | **Base-**  **pairing**  **(bp)** | **Template dependency** | **Average**  **yield (%)** | **Reaction Conditions** |
| --- | --- | --- | --- | --- | --- | --- | --- |
| **#1** | Duplex | **/** | C12, C6 | 20 | P | 87.4 (C12) | 37 °C, 72 h |
| **#2** | Blunt-Y | **/** | C6 | 14+14+3 | P | 68.8 | 37 °C, 72 h |
| **#3** | Overhang-Y | **/** | C6 | 14+14+3 | P | 63.4 | 37 °C, 72 h |
| **#4** | T-motif | **/** | C6 | 5 | I | 20.6 | 20 °C, 72 h |
| **#5** | T-motif | **/** | C6 | 5, 6 | I | 35.6 (6 bp)  24.8 (5 bp) | 20 °C, 72 h |
| **#6** | Overhang-Y | **=** | C6 | 14+14+3 | P | 30.3 | 37 °C, 48 h |
| **#7** | Linear | **/** | C12 | 16+16 | P | 55.4 | 37 °C, 48 h |
| **#8** | Mid-overhang | **/** | C6 | 16+16 | P | 45.4 | 37 °C, 48 h |
| **#9** | 3WJ | **/** | C6 | 15+15 | P | 49.1 | 37 °C, 48 h |
| **T-motif Tile** | Dual T-motifs | **=** | C6 | 6 | I | ND | 20 °C, 72 h |

/ : separated strand; = : both group in a strand; P : proportion; I : inverse proportion; ND : no data

**References**

1. Suzuki, T., Ide, H., Yamada, M., Endo, N., Kanaori, K., Tajima, K., Morii, T., Makino, K. (2000) Formation of 2'-deoxyoxanosine from 2'-deoxyguanosine and nitrous acid: mechanism and intermediates. Nucleic Acids Res, 28, 544-551.
2. Bohlin, J., Matthies, M., Poppleton, E., Procyk, J., Mallya, A., Yan, H., and Šulc, P. (2022) Design and Simulation of DNA, RNA, and Hybrid Protein-Nucleic Acid Nanostructures with oxView. Nat Protoc, 17, 1762-1788.
3. Meng, E.C., Goddard, T.D., Pettersen, E.F., Couch, G.S., Pearson, Z.J., Morris, J.H. and Ferrin, T.E. (2023) UCSF ChimeraX: Tools for Structure Building and Analysis. Protein Sci, 32, e4792.
4. Fantoni, N.Z., El-Sagheer, A.H., and Brown, T. (2021) A Hitchhiker’s Guide to Click-Chemistry with Nucleic Acids. Chem. Rev., 121, 7122–7154.
5. Hennessy, J., McGorman, B., Molphy, Z., Farrell, N.P., Singleton, D., Brown, T., and Kellett, A. (2022) A Click Chemistry Approach to Targeted DNA Crosslinking with cis-Platinum(II)-Modified Triplex-Forming Oligonucleotides. Angew. Chem. Int. Ed., 61, e202110455.
6. Basu, S., Roy, A., Barcenas, G., Li, X., Yurke, B., Knowlton, W.B., and Lee, J. (2023) Enhanced Photo-crosslinking of Thymines in DNA Holliday Junction-Templated Squaraine Dimers. Biochemistry, 62, 3234–3244.
7. Rajendran, A., Endo, M., Katsuda, Y., Hidaka, K., and Sugiyama, H. (2011) Photo-Cross-Linking-Assisted Thermal Stability of DNA Origami Structures and Its Application for Higher-Temperature Self-Assembly. J. Am. Chem. Soc., 133, 14488–14491.
